# Mass spectrometric methods for monitoring redox processes in electrochemical cells

**DOI:** 10.1002/mas.21409

**Published:** 2013-12-10

**Authors:** Herbert Oberacher, Florian Pitterl, Robert Erb, Sabine Plattner

**Affiliations:** 1 Institute of Legal Medicine and Core Facility Metabolomics, Innsbruck Medical UniversityInnsbruck, Austria

**Keywords:** electrochemistry, mass spectrometry, liquid chromatography, electrospray ionization, electron ionization, differential electrochemical mass spectrometry

## Abstract

Electrochemistry (EC) is a mature scientific discipline aimed to study the movement of electrons in an oxidation–reduction reaction. EC covers techniques that use a measurement of potential, charge, or current to determine the concentration or the chemical reactivity of analytes. The electrical signal is directly converted into chemical information. For in-depth characterization of complex electrochemical reactions involving the formation of diverse intermediates, products and byproducts, EC is usually combined with other analytical techniques, and particularly the hyphenation of EC with mass spectrometry (MS) has found broad applicability. The analysis of gases and volatile intermediates and products formed at electrode surfaces is enabled by differential electrochemical mass spectrometry (DEMS). In DEMS an electrochemical cell is sampled with a membrane interface for electron ionization (EI)-MS. The chemical space amenable to EC/MS (i.e., bioorganic molecules including proteins, peptides, nucleic acids, and drugs) was significantly increased by employing electrospray ionization (ESI)-MS. In the simplest setup, the EC of the ESI process is used to analytical advantage. A limitation of this approach is, however, its inability to precisely control the electrochemical potential at the emitter electrode. Thus, particularly for studying mechanistic aspects of electrochemical processes, the hyphenation of discrete electrochemical cells with ESI-MS was found to be more appropriate. The analytical power of EC/ESI-MS can further be increased by integrating liquid chromatography (LC) as an additional dimension of separation. Chromatographic separation was found to be particularly useful to reduce the complexity of the sample submitted either to the EC cell or to ESI-MS. Thus, both EC/LC/ESI-MS and LC/EC/ESI-MS are common.

## I. INTRODUCTION

Electrochemistry (EC) is regarded as a mature scientific discipline, having a distinguished 200-year-old history. Combination of advances in material sciences, electronics, computing, mathematics, physics, chemistry, and the biological sciences have enabled the construction of convenient electrochemical packages for diverse fields of application. Batteries, fuel cells and photovoltaic cells are important devices for production and storage of energy. Important materials, including aluminum, copper and zinc, are produced by electrorefining techniques. Electrochemical sensors are commonly used medical devices to monitor for instance glucose in diabetes patients. Corrosion also represents an important example of an electrochemical process with considerable impact on modern society and economy.

Given the significance of electrochemical technology and the widespread teaching of the basics of electrochemistry in undergraduate university and senior science courses, it could be logically assumed that most scientists have an excellent understanding of the theoretical and experimental aspects of the subject. Accordingly, only a brief summary of the most important concepts is provided in this review.

The fundamental of EC is the study of the movement of electrons in an oxidation–reduction reaction. EC covers techniques that use a measurement of potential, charge, or current to determine the concentration or the chemical reactivity of analytes. To understand EC five important and interrelated concepts need to be appreciated: (1) the electrode's potential determines the analyte's form at the electrode's surface; (2) the concentration of the analyte at the electrode's surface may not be the same as its concentration in bulk solution; (3) in addition to an oxidation–reduction reaction, the analyte may participate in other reactions; (4) current is a measure of the rate of the analyte's oxidation or reduction; and (5) current and potential cannot be controlled simultaneously.

**Figure 1:**
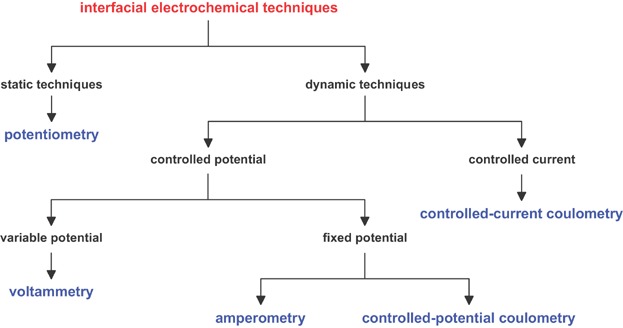
The family tree of interfacial electrochemical techniques.

Electrochemical measurements are made in an electrochemical cell consisting of two or more electrodes. Electroanalytical methods can be broken down into several categories depending on which aspects of the cell are controlled and which are measured. Bulk electrochemical techniques are usually based on the measurement of a solution's conductivity. The four main categories of interfacial electrochemical techniques are potentiometry, coulometry, amperometry, and voltammetry (Fig. 1).

**Figure 2:**
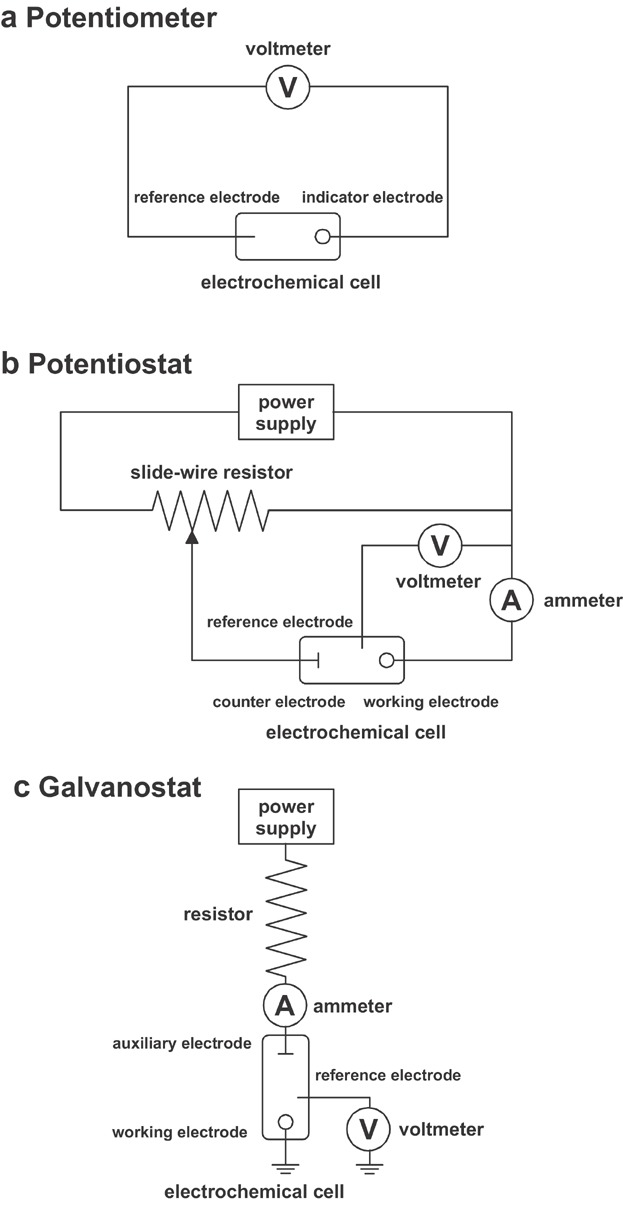
Schematic diagrams of (a) a potentiometer, (b) a potentiostat, and (c) a galvanostat.

In potentiometry the potential of an electrochemical cell under static conditions is measured. Because no or only a negligible current flows through the electrochemical cell, its composition remains unchanged. The Nernst equation relates an electrochemical cell's potential to the concentration of electroactive species. A typical setup of a potentiometer is shown in Figure 2a.

In coulometry the analyte is completely converted from one oxidation state to another or it reacts completely with a reagent generated at the working electrode. The Faraday's law relates the total charge passing through the cell to the absolute amount of electroactive species. There are two forms of coulometry: controlled- potential coulometry, in which a constant potential is applied to the electrochemical cell (Fig. 2b), and controlled-current coulometry, in which a constant current is passed through the electrochemical cell (Fig. 2c).

In amperometry a constant potential is applied to the working electrode (Fig. 2b). The current is measured as a function of time and related to the concentration of the analyte. One important application of amperometry is in the construction of chemical sensors (e.g., sensors for glucose or O_2_).

**Figure 3:**
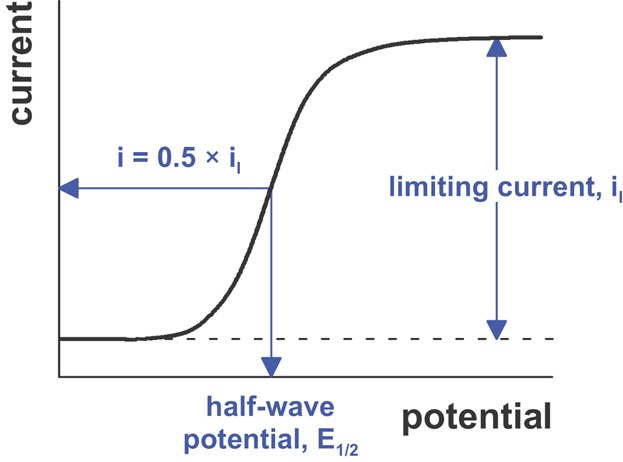
Voltammogram including the limiting current (*i*_l_) and the half-wave potential (*E*_1/2_).

In voltammetry a time-dependent potential is applied to an electrochemical cell and the resulting current is measured as a function of that potential. Although early voltammetric methods used only two electrodes, a modern voltammeter makes use of a three-electrode potentiostat (Fig. 2b). In such a setup, the potential at the working electrode is changed relative to the fixed potential of the reference electrode and the faradaic current is measured that flows between the working and auxiliary electrodes. The resulting plot of current versus applied potential is called a voltammogram (Fig. 3). A voltammogram is providing quantitative and qualitative information about the species involved in the oxidation or reduction reaction. The shape of a voltammogram is determined by several experimental factors, including standard potentials of electroactive species, mass transport effects, electron transfer kinetics, and secondary chemical reactions. The half-wave potential is closely related to the standard potential but is usually not identical to it. Thus, half-wave potentials are sometimes useful for identification of components of a solution. The limiting current or peak current is generally directly proportional to the analyte's concentration in the bulk solution and can, therefore, be used for quantitative analysis.

EC represents a fast, simple, and inexpensive technology with widespread application in qualitative and quantitative analysis. From the analytical point of view, the best feature of EC is the direct conversion of chemical information into an electrical signal. Despite considerable success, however, pure electroanalytical methods may suffer from the problem that compound identification is based on measured electrochemical potentials only. Often increased identification power is required for in-depth characterization of complex electrochemical reactions involving the formation of diverse intermediates, products, and byproducts. Accordingly, combinations of EC with alternative analytical techniques enabling the off-line or on-line characterization of electrochemical reactions are gaining more and more attention. Particularly mass spectrometry (MS) has found broad applicability in this context.

EC is usually performed in solution. To enable mass spectrometric monitoring of these processes, analytes need to be transferred into the gas-phase and ionized. For the analysis of very volatile compounds, differential electrochemical mass spectrometry (DEMS) has found broad applicability. A hydrophobic membrane is used as interface between EC and MS, and electron ionization (EI) is employed. For the mass spectrometric analysis of other molecules, several different ionization techniques were in use (e.g., thermospray ionization, particle beam ionization). Today, however, electrospray ionization (ESI) is the most important ionization technique in EC/MS. ESI-MS allows the sensitive and comprehensive characterization of products formed by electrochemical reactions. For the analysis of very complex samples, EC/ESI-MS is usually complemented by liquid chromatography (LC).

Over the years a number of reviews have been published summarizing different aspects of the hyphenation of EC to MS (Mora et al., 2000; Diehl & Karst, 2002a; Baltruschat, 2004; Karst, 2004; Roussel et al., 2004; Van Berkel & Kertesz, 2007; Lohmann & Karst, 2008; Permentier, Bruins, & Bischoff, 2008; Abonnenc et al., 2010; Baumann & Karst, 2010; Gun et al., 2010; Roeser et al., 2010a; Faber et al., 2011; Nouri-Nigjeh et al., 2011a; Jahn & Karst, 2012). The present review is intended to give an overview on instrumental designs currently used for EC/MS and on the experimental conditions essential for successful hyphenation of EC to MS. DEMS will be the main topic of the first part of this review. Next, a description of the inherent EC of ESI will be provided. Finally, different combinations of EC with ESI-MS and LC/ESI-MS will be discussed.

## II. DIFFERENTIAL ELECTROCHEMICAL MASS SPECTROMETRY—EC/EI-MS

### A. Introduction to DEMS

The first attempts of on-line monitoring electrochemical reactions with MS date back to the 1970s. In their pioneering work Bruckenstein and Gadde used MS to detect gaseous products (O_2_ and H_2_) generated in an electrochemical cell containing perchloric acid (Bruckenstein & Gadde, 1971). With the help of a hydrophobic membrane directly connected to a porous electrode gaseous products were selectively transferred from the electrochemical cell into the mass spectrometric system. The typical response time of that initial system was 20 sec. The method was enhanced by Wolter and Heitbaum (1984). They considerably improved the vacuum system so that the response time became short enough (<1 sec) to allow the on-line detection of reaction products during cyclic voltammetry. The method was called DEMS. The term “differential” was used to emphasize that in contrast to the earlier method this technique provided kinetic information (Wolter & Heitbaum, 1984). Others refer “differential” to the use of a two-stage pressure reduction system in the mass spectrometer (“differential pumping”) (Tsiouvaras et al., 2013).

**Figure 4:**
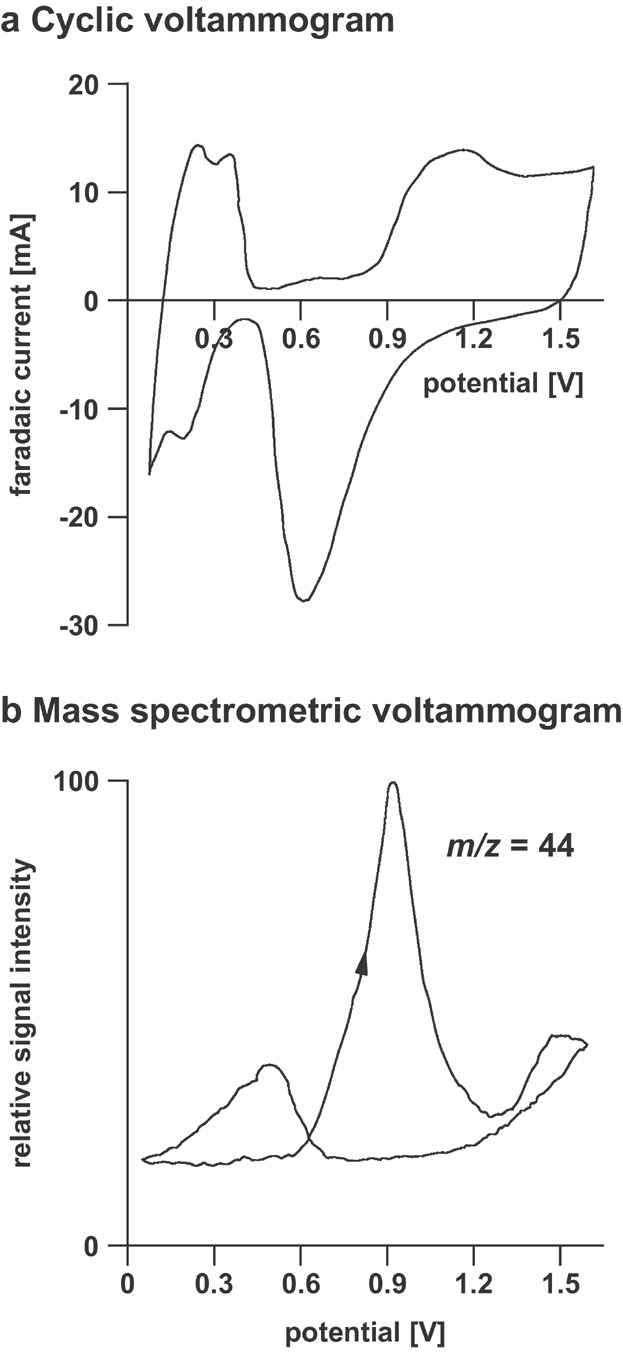
(a) Cyclic voltammogram and (b) mass spectrometric voltammogram of CO_2_ at a Pt-electrode in 0.5 M H_2_SO_4_ and 2.5 mM methanol (sweep rate 400 mV sec^−1^). Reproduced from Wolter and Heitbaum (1984) with permission of the “Deutsche Bunsen-Gesellschaft für Physikalische Chemie” (Copyright 1984).

DEMS has proven a highly valuable method for the direct, qualitative, and quantitative measurement of dissolved gases and volatile intermediates and products formed at electrode surfaces during potential sweep, potentiostatic, and galvanostatic experiments (Baltruschat, 2004; Gun et al., 2010). In a typical DEMS experiment, the ion current of one or more species involved in an electrochemical reaction are selectively measured as function of time or varying electrode potential. Plots of intensity versus electrochemical potential are called mass spectrometric voltammograms. An example of a mass spectrometric voltammogram is shown in Figure 4. In this experiment methanol oxidation on Pt in sulfuric acid was studied by cyclic voltammetry employing a sweep rate of 400 mV sec^−1^ (Wolter & Heitbaum, 1984). The faradaic current and the ion current of CO_2_ (*m*/*z* = 44) were monitored in parallel.

A DEMS instrument for on-line monitoring of electrochemical processes should have low limits of detection, fast response times, and linear proportionality between concentration and ion current. Today, there is no commercial DEMS instrument available. The technique has mainly found applicability in research laboratories. Common fields of application have been reviewed by Baltruschat (2004). Particularly, oxidation reactions occurring in fuel cells have extensively been studied with DEMS (Wolter, Willsau, & Heitbaum, 1985; Ianniello & Schmidt, 1995; Wasmus, Wang, & Savinell, 1995; Jambunathan, Jayaraman, & Hillier, 2004; Seiler et al., 2004; Housmans, Wonders, & Koper, 2006; Chojak Halseid, Jusys, & Behm, 2010; Wang, Rus, & Abruna, 2010).

### B. DEMS Instrumentation

The mass spectrometric part of the DEMS system usually consists of a quadrupole instrument employing EI. The differentially pumped vacuum system consists of at least two, individually pumped vacuum chambers that are connected by a small aperture or skimmer. The ion source is usually located between the first and the second pumping stage.

The electrochemical part is separated from the mass spectrometric part by a hydrophobic membrane. Thus in some respect, DEMS resembles membrane introduction MS (Kotiaho et al., 1991). The hydrophobic nature of the membrane prevents extensive transfer of electrolyte into the mass spectrometer. Principally, only dissolved gaseous, volatile and relatively non-polar species are allowed to evaporate into the vacuum system. Porous membranes are typically made of polytetrafluoroethylene (Gore-Tex, 75 µm thick, 50% porosity, 0.02 µm pore diameter) or ethylene-tetrafluoroethylene copolymer (Scimat, 60 µm thick, 50% porosity, 0.2 µm pore diameter). These materials are known to be durable and chemically resistant. The membranes are often supported by stainless steel or glass frits to increase their mechanical stability. The transport of compounds through the membrane pores is via the gas phase. Due to the use of membranes with narrow pore radii, the transport of the non-wetting aqueous electrolyte through the pores is prevented. A significant quantity of water, however, enters the mass spectrometer via the gas phase owing to the pressure drop across the membrane. Consequently, a reasonably sized vacuum system is obligatory to maintain an operating pressure (<10^−5^ mbar) necessary for reaching low limits of detection. To reduce the leakage of water, capillary inlets were developed (Gao et al., 1994; Jambunathan & Hillier, 2003; Jambunathan, Jayaraman, & Hillier, 2004; Wonders et al., 2006). Alternatively, the use of a nonporous polymer membrane was proposed (Skou & Munk, 1994). There, the transport is accomplished by diffusion through the membrane only. A drawback of this approach is the observed increase of response time.

#### 1. The Conventional DEMS Interface

**Figure 5:**
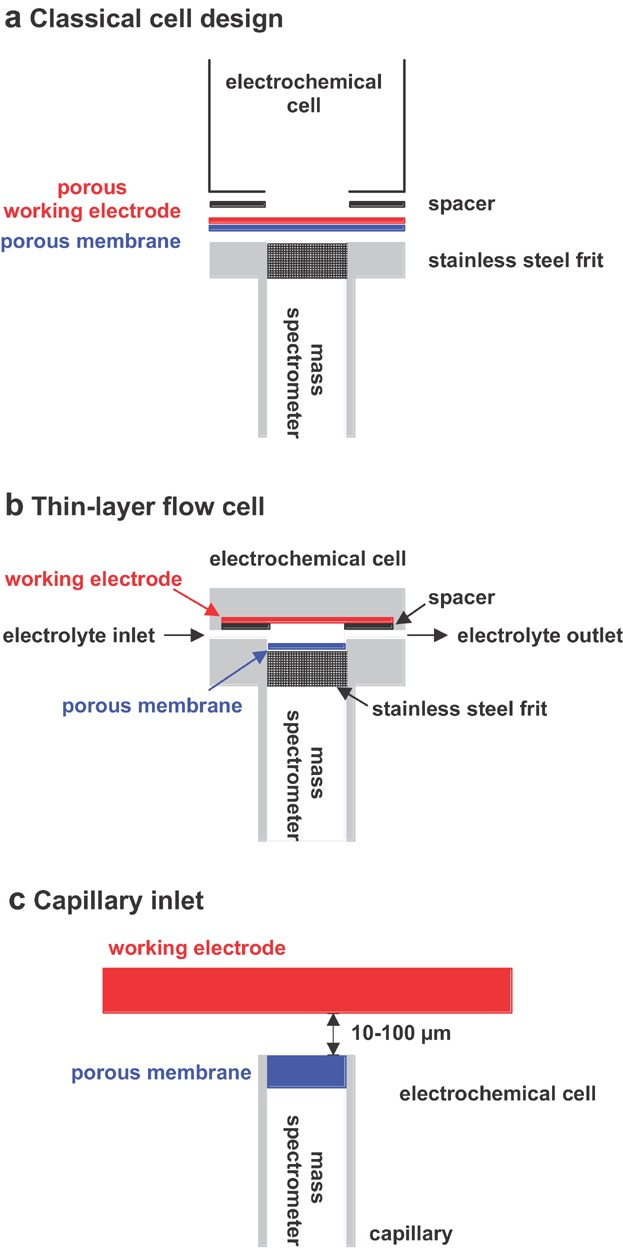
Illustration of (a) the classical cell design, (b) the thin-layer flow cell, and (c) the capillary inlet used for DEMS.

A schematic drawing of the conventional DEMS interface is shown in Figure 5a. The porous working electrode (WE) is directly linked to the porous membrane interface. Such electrodes are prepared either by sputter deposition of an electrocatalyst layer or by painting a lacquer containing small metallic particles (Baltruschat, 2004). This design is characterized by fast response times (<1 sec) and high collection efficiencies. Disadvantages of this setup include depletion of volatile reactants, not well-defined mass transport, and the inability to use massive electrodes.

Rotating systems were developed to enhance mass transport (Tegtmeyer, Heitbaum, & Heindrichs, 1989; Wasmus, Cattaneo, & Vielstich, 1990; Fujihira & Noguchi, 1993). Tegtmeyer, Heitbaum, and Heindrichs (1989) adopted a rotating porous electrode as an inlet system to the mass spectrometer. Wasmus, Cattaneo, and Vielstich (1990) positioned a rotating cylinder electrode near the inlet membrane window to the mass spectrometer. Fujihira and Noguchi (1993) placed a rotating rod above the stationary gas permeable porous electrode.

#### 2. Thin-Layer Cells

Thin-layer cells were developed for applying smooth or crystalline electrodes (Hartung & Baltruschat, 1990; Hartung et al., 1991). As shown in Figure 5b, the WE is separated from the membrane interface via a thin-layer of electrolyte. The distance between the WE and the membrane interface is ascertained by a spacer made of Teflon (50 µm thick) giving a cell volume of a few microliters. Electrochemical reaction products are transported from the electrode surface to the membrane by diffusion, which may lead to increased response times (2–3 sec) and reduced collection efficiency particularly under continuous flow conditions.

A modified thin-layer cell was developed by Bogdanoff, Friebe, and Alonso-Vante (1998). In their setup a ring of a gas-permeable membrane is pressed upon the electrode. This ring separates the high vacuum from the electrolyte. Volatile reaction products diffuse sideways through the membrane into the mass spectrometer where they are analyzed and detected.

Thin-layer cells have found to be useful to couple DEMS with other analytical techniques. Such hyphenated techniques have the vast potential to provide new insights into the kinetics and mechanisms of electrochemical reactions. Jusys, Massong, and Baltruschat (1999) have presented a dual tape thin-layer flow-through cell for simultaneous measurements with DEMS and an electrochemical quartz crystal microbalance (EQCM). EQCM is a technique for the in situ measurement of the electrode mass change by the change of the oscillation frequency of the quartz crystal due to deposition or dissolution of the solid phase during faradaic processes. The setup was applied to study CO oxidation on Pt. The combination of DEMS and in situ attenuated total reflection-infrared spectroscopy (ATR-FTIRS) was enabled by a dual thin-layer cell (Heinen et al., 2007). The potential of this setup was demonstrated by studying CO oxidation on Pt. In a cell design introduced by Wang, Rus, and Abruna (2010) a double-band-electrode flow-through cell was used to combine DEMS with the subsequent electrochemical detection of nonvolatile products. The cell consisted of two band electrodes, which served as working and detecting electrodes, respectively, separated by a porous Teflon membrane acting as interface to the mass spectrometer. The setup was used to study the electrooxidation of formaldehyde and methanol on carbon supported Pt nanoparticle catalysts.

#### 3. Miniaturized Interfaces

**Figure 6:**
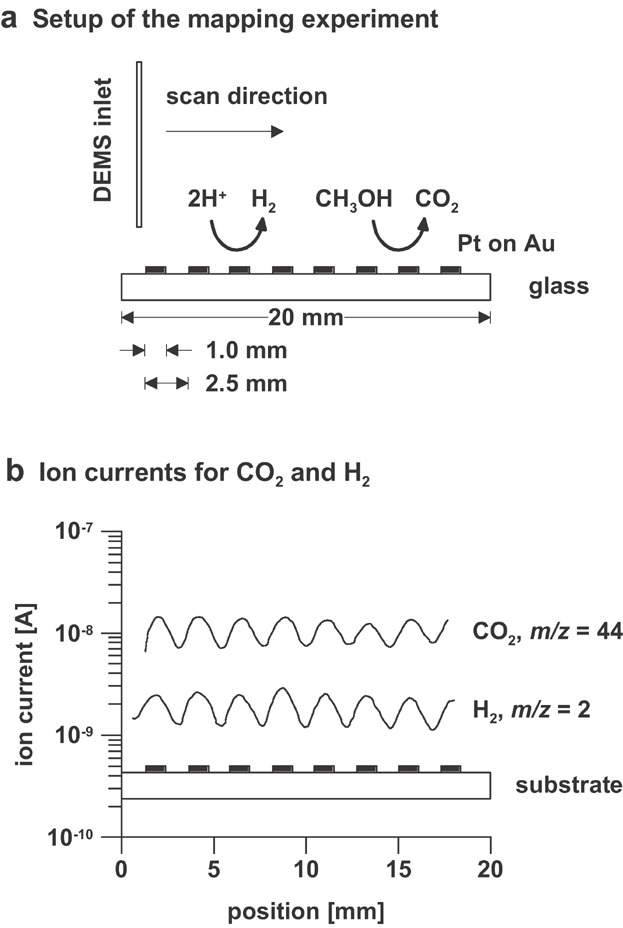
Spatial mapping of a band electrode assembly by capillary DEMS. Details on (a) the setup of the mapping experiment and (b) the measured ion currents for CO_2_ and H_2_ are provided. The bands comprised vapor deposited Au on glass with a layer of electrodeposited Pt. For H_2_ evolution, the substrate was held at a constant potential of −0.05 V versus the reversible hydrogen electrode (RHE). For methanol oxidation, the substrate was held at a constant potential of 0.5 V versus RHE. Both measurements were performed in a solution containing 0.5 M H_2_SO_4_ and 1 M methanol using a raster rate of 25 µm sec^−1^. Reproduced from (Jambunathan & Hillier, 2003) with permission of the Electrochemical Society (Copyright 2003).

Gao et al. introduced another setup to study electrochemical processes at massive electrodes (Gao et al., 1994). They constructed a miniaturized interface for sampling reaction products over a very small fraction of the electrode surface. The interface consisted of a pin-hole several micrometer in diameter located at the center of the hemispherical end of a thick glass tube several mm in diameter. The pin-hole was covered with a Teflon film (50 µm thick) and was brought in close proximity (a few micrometer) of the electrode. An improved version of miniaturized interface was presented recently (Jambunathan & Hillier, 2003; Jambunathan, Jayaraman, & Hillier, 2004; Wonders et al., 2006; Roos et al., [Bibr b175],[Bibr b176]; Rus et al., 2013). The porous membrane was incorporated into the end of a capillary (0.15–0.6 mm inner diameter), and a three-dimensional positioning system was used to bring the capillary in close proximity (10–100 µm) of the electrode (Fig. 5c). A clear disadvantage of the miniaturized system is the rather long response time. Furthermore, as a larger portion of the solution than the cylindrical volume between the capillary and the electrode is sampled, absolute quantitation is difficult. The main advantage of the miniaturized system is that, due to the use of a small gas inlet leading to an inherently lower gas flux, a simpler vacuum system can be used, thereby considerably reducing the detection limit of the mass spectrometric system. Furthermore, the miniaturized system can be used to scan electrode surfaces (Jambunathan & Hillier, 2003; Jambunathan, Jayaraman, & Hillier, 2004). By changing the position of the capillary inlet, the electrochemical formation of specific products can be measured at defined locations on an electrode. For high spatial resolution the inlet of the mass spectrometer comprised a capillary with an inner diameter of 150 µm, and the tip-substrate gap was reduced to less than 100 µm. An example for the spatial mapping capabilities of a miniaturized DEMS system is shown in Figure 6. The substrate comprised a series of band electrodes of 1 mm width and 2.5 mm period. The bands comprised vapor deposited gold on glass with a layer of electrodeposited platinum at the outer surface. The substrate was placed in a aqueous solution containing 1.0 M CH_3_OH and 0.5 M H_2_SO_4_ in order to sample, in sequential measurements, the products of methanol oxidation and hydrogen evolution. During these experiments, the Pt bands were shorted to each other and held at the same potential.

### C. DEMS Applications

DEMS is a commonly applied method to monitor small volatile molecules representing educts, intermediates or products of processes occurring in electrochemical cells. Particularly, CO, CO_2_, H_2_, and O_2_ are common targets. Important applications of DEMS are summarized in Table1. DEMS experiments are particularly useful to study and to optimize the EC of batteries, fuel cells, and photovoltaic cells.

**Table 1:** Important applications of DEMS

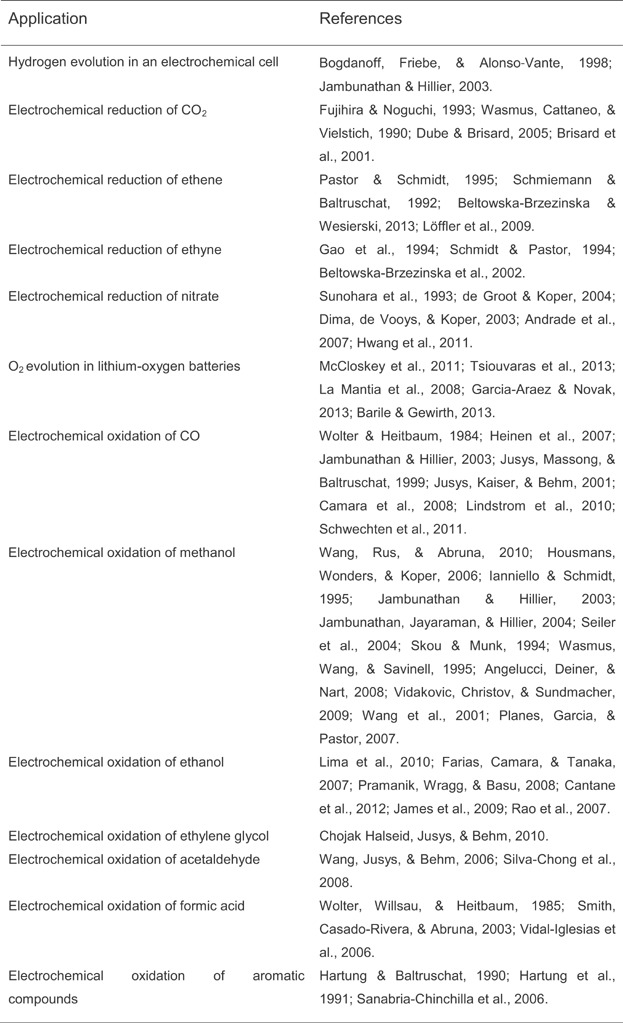

## III. THE ELECTROCHEMICAL PART OF ELECTROSPRAY IONIZATION

ESI-MS has been developed by Fenn and coworkers in the 1980s (Yamashita & Fenn, 1984; Whitehouse et al., 1985; Meng, Mann, & Fenn, 1988), and represents a versatile analytical tool for the characterization of small to large molecules and their complexes (Cole, 1997). ESI was found to be particularly useful for the analysis of (bio-)organic molecules, including peptides, proteins and nucleic acids, and allows the direct hyphenation of separation techniques such as LC and capillary electrophoresis with MS.

### A. The ESI Mechanism

In ESI an analyte solution is injected through a capillary emitter at flow rates ranging from a few nanoliters per minute to several hundred microliters per minute. A high voltage (2–6 kV) is applied to the capillary relative to a counter electrode (i.e., the mass spectrometer). This strong electric field causes the dispersion of the sample solution into an aerosol of highly charged droplets, leading to the formation of gas-phase ions. ESI can be operated in positive or negative ion mode, wherein a positive or negative potential difference induces the formation of gas-phase cations or anions, respectively.

Profound understanding of the fundamentals of ESI is considered to be of importance for improving performance, to expand the range of analytes amenable to analysis, and to control or alter the ionic species observed. Over the years mechanistic aspects of the ESI process were extensively studied and often controversially discussed (Mora et al., 2000). Today there is agreement that ESI involves three main steps before analysis—generation and charging of droplets; droplet evaporation and production of gas-phase ions; and secondary processes that modify the gas-phase ions in the atmospheric and subatmospheric-pressure sampling regions of the mass spectrometer (Van Berkel & Kertesz, 2007).

### B. EC in the ESI Emitter

The electrochemical aspects of ESI have been extensively studied (Mora et al., 2000; Van Berkel & Kertesz, 2007; Girault et al., 2010). Thus, practitioners have learned to control the electrochemical part of this technique in a way that the impact of EC on the mass spectra observed can be very much tuned. In the majority of cases experimental conditions are chosen that prevent the mass spectrometric detection of electrochemical reactions. In some situations, however, EC can be used to analytical advantage, and these cases will be summarized in the following.

**Figure 7:**
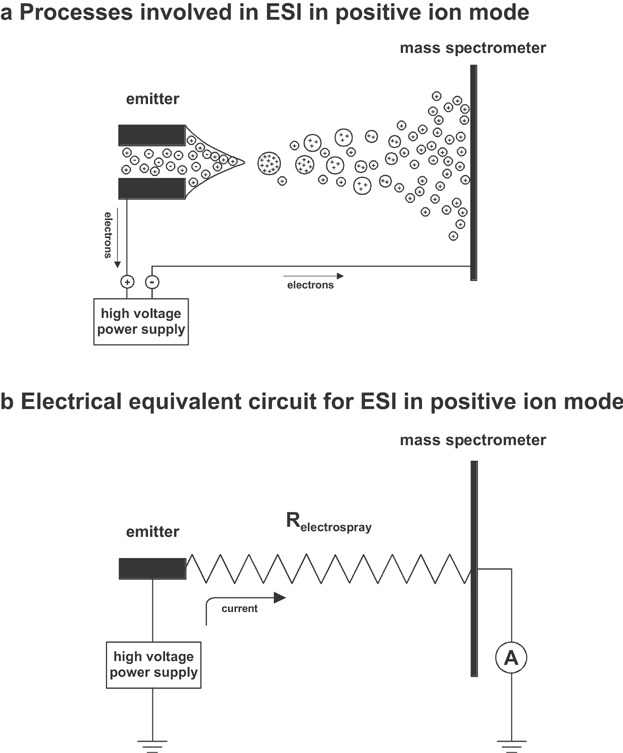
Schematic representations of (a) the processes that are assumed to occur in ESI in positive ion mode and of (b) the electrical equivalent circuit for the ESI source in positive ion mode. Reproduced from Blades, Ikonomou, and Kebarle (1991) with permission of the American Chemical Society (Copyright 1991) and from Van Berkel et al. (2004b) with permission of the Springer-Verlag (Copyright 2004).

EC was first identified an important part of the ESI process by Blades, Ikonomou, and Kebarle (1991) and Van Berkel, McLuckey, and Glish (1991, 1992). From the electrochemical point of view (Van Berkel & Zhou, 1995a; Jackson & Enke, 1999), the ESI source represents a controlled-current cell consisting of two electrodes (Fig. 7). One electrode is the capillary emitter; the mass spectrometer acts as the counter electrode. The two electrodes are connected on the one hand by the power supply and on the other hand by a series of resistors consisting of the electrochemical contact to the solution, the solution resistance, the resistance at the solution–air interface and in the gas-phase, and the charge neutralization at the counter electrode. Usually, the solution–air interface resistance and the gas-phase resistance limit the faradaic current.

The electrochemical contact occurs between the metal to which the power supply is connected and the solution in that region of the metal-solution contact closest to the electrospray tip. If the connection is to a metallic union and a non-metallic glass capillary is used as the spray needle, there may be some solution resistance between the electrochemical contact and the spray tip. At the needle tip, charge separation occurs as a result of the high electric field that exists between the tip and the counter electrode. The charge separation is in the formation of the charged droplets that emanate from the tip. The charged droplets are then attracted across the air gap between the tip and the counter electrode. All of the charge that is separated at the tip is neutralized at the counter electrode or inside the mass spectrometer and returned to the power supply.

As outlined by Blades, Ikonomou, and Kebarle (1991) and by Van Berkel and Zhou (1995a), the electrophoretic charge separation of ions in the solution at the emitter tip induced by the electric field is leading to selective loss of ion polarity in the droplets and to accumulation of ions of the opposite polarity in the solution. This build-up of charge would create an electric field counteracting the externally applied field, which would finally quench charged droplet formation. Therefore, any charge transported via the gas-phase to the mass spectrometer needs to be balanced in solution. The charge-balancing is accomplished by electrochemical processes at the metal-solution interface. The electrochemical reactions at the metal-solution interface may involve compounds dissolved in the solvent, the solvent itself, and the electrode material. The resulting interfacial potential will be at or near the electrochemical potential of those reactions that sufficiently supply all the required current.

#### 1. Corrosion of the Electrode Material

In positive ion mode, the electrode material may undergo anodic corrosion, thus liberating metal ions into the solution. Corrosion of electrode material was first reported by Blades, Ikonomou, and Kebarle (1991). When Zn, which is a very easy to oxidize metal, was used as emitter material, Zn^2+^ ions were detected in the sprayed solution. Similar results were obtained when using stainless steel emitters. In this case Fe^2+^ ions were released. A more detailed study on the oxidation reactions involving stainless steel emitters was presented by Van Berkel (1998). He integrated a photodiode array detector between the electrochemical contact and the spray tip. He attempted to detect Fe^2+^ ions as tris(1,10-phenanthroline)iron(II), and Fe^3+^ ions as Fe(SCN)_3_. However, only the production of Fe^2+^ ions was observed.

The corrosion of several different metal electrode materials was studied by Van Berkel, Asano, and Schnier (2001). The electrode materials tested included Pt, stainless steel, Fe, and Cu. 1,10-Phenanthroline was used as indicator for the production of metal ions in a wire-in-a-capillary nano-electrospray emitter. This compound forms stable complexes with metal ions that can be detected by MS. When using the Pt electrode, there was no evidence for the occurrence of electrochemical corrosion. With all other electrode materials tested, however, phenantroline complexes were observed that indicated the formation of Fe^2+^, Fe^3+^, Cu^+^, or Cu^2+^ ions.

Corrosion events can have substantial analytical consequences. Usually, the production of metal ions may result in increased chemical noise, and should therefore be avoided. Electrode corrosion has been used for redox buffering (Van Berkel & Kertesz, 2001; Peintler-Krivan, Van Berkel, & Kertesz, 2010a,2010b). By employing emitter materials with low redox potentials (i.e., Cu, poly(pyrrole) polymer film) the interfacial potential is maintained near the equilibrium potential of the corrosion process, and thus control over the electrochemical reactions that take place at this electrode is provided. The utility of this approach was demonstrated by completely avoiding the oxidation of N-phenyl-1,4-phenylenediimine with a Cu emitter in comparison to a stainless steel emitter (Van Berkel & Kertesz, 2001), as well as by completely avoiding the oxidation of amodiaquine and reserpine with a polymer-coated electrode in comparison to a bare metal emitter electrode (Peintler-Krivan, Van Berkel, & Kertesz, 2010a,2010b).

There are situations where the involvement of corrosion processes is beneficial. Anodic corrosion has been utilized to supply metal ions to solution for the study of metal-ligand complexes chemistry or to ionize particular molecules by metal-analyte cationization. Girault and coworkers made use of Cu and Zn electrodes to produce Cu^+^/Cu^2+^ and Zn^2+^ ions for complex formation with small molecules (Prudent, Roussel, & Girault, 2007) and peptides (Rohner & Girault, 2005; Lu et al., 2010; Prudent, Roussel, & Girault, 2007; Prudent & Girault, [Bibr b163],[Bibr b164]). This method avoids the addition of metallic salts, therefore preventing signal suppression induced by anions introduced together with the metal ions (Van Berkel, Asano, & Schnier, 2001), and allows the efficient production of short-lived metal ions (i.e., Cu^+^) for in situ tagging reactions.

#### 2. EC of Solvent Additives

Redox reactions that take place in the emitter capillary of an ESI source may alter the composition of the solvent. A well-known example of solution composition change is the alteration of the pH (Van Berkel, Zhou, & Aronson, 1997; Konermann, Silva, & Sogbein, 2001; Pan et al., 2004). Anodic oxidation of water in positive ion mode can decrease the pH of the initial solution; reduction in negative ion mode will lead to an increase of the pH. Depending on the experimental conditions used, solution pH may change by 4 pH units. The magnitude of the pH change will be most significant for non-buffered solutions near neutral pH when using low-flow-rate ESI-MS systems (Van Berkel, Zhou, & Aronson, 1997). The alteration of the pH can have an impact on the mass spectra observed by changing the equilibrium ion distribution of basic or acidic analytes, and this has been demonstrated for different proteins (Van Berkel, Zhou, & Aronson, 1997; Konermann, Silva, & Sogbein, 2001; Pan et al., 2004).

Additives may be added to the solvent to induce homogenous redox buffering. Such compounds “buffer” the potential to a level near the equilibrium potential for its redox reaction. Efficient redox buffers should more easily undergo electrolysis at the emitter electrode than analytes and neither the original compound nor its electrolysis product(s) should be detectable directly or indirectly by ESI-MS. Homogenous redox buffer systems presented include iodide (Van Berkel, Zhou, & Aronson, 1997), hydroquinone (Moini, Cao, & Bard, 1999), and ascorbic acid (Plattner et al., 2012a).

**Figure 8:**
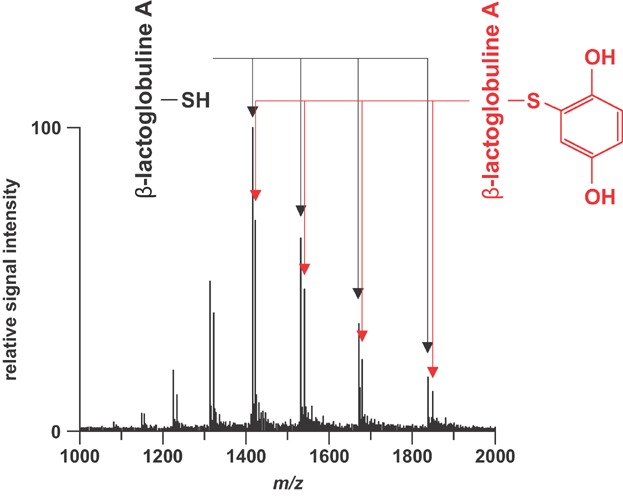
Mass spectrum of β-lactoglobuline A infused with 5 mM p-hydroquinone through a nanospray interface. Doublet peaks appear corresponding to the distribution of native and modified protein. This spectrum provides the number of tagged cysteines, in this case one because only the native plus one shifted peak are observable per charge state. Reproduced from Rohner, Rossier, and Girault (2002) with permission of Elsevier Science B.V. (Copyright 2002).

EC of solvent additives can be used to generate probes for subsequent homogenous reaction with analytes. Girault and coworkers have developed a technique for nonquantitative mass tagging of cysteine residues in peptides and proteins (Rohner, Rossier, & Girault, 2002; Roussel et al., [Bibr b177],[Bibr b178]; Dayon, Josserand, & Girault, 2005; Dayon, Roussel, & Girault, 2006; Girault et al., 2010). Mixtures of the analyte and a hydroquinone are infused through a microspray emitter. At the emitter electrode, the hydroquinone is oxidized to the corresponding benzoquinone which undergoes a 1,4-Michael addition of cysteine. MS analysis enables the simultaneous detection of unmodified and modified species (Fig. 8). The number of characteristic mass shifts observed corresponds to the number of cysteine residues in the peptide/protein. The determination of the cysteine content was found to be beneficial for unequivocal protein identification in peptide fingerprinting experiments.

#### 3. EC of Analytes

Electrochemical reactions directly involving analytes are usually suppressed by selecting proper experimental conditions. Their occurrence, however, cannot fully be excluded (Paim et al., 2005; Weber, Von Wiren, & Hayen, 2006; Wu et al., 2009; Raji, Amad, & Emwas, 2013). Redox reactions do not only occur at the emitter electrode, gas phase reactions might also be responsible for observed alterations (Gianelli et al., 2001; Boys et al., 2009).

Electrochemical reactions directly involving analytes can also be used to analytical advantage. Usually, ESI is best suited to analyze compounds that are already ionized in solution. Under typical operating conditions favoring protonation, deprotonation, or ion attachment, ESI is an inefficient ionization method for neutral, low-polarity compounds. By making use of the inherently present EC, however, the utility of ESI can be expanded to metalloporphyrins, polycyclic aromatic hydrocarbons, aromatic amines, heteroaromatics, ferrocenes, fullerenes, polyenes, and quinones (Van Berkel, McLuckey, & Glish, 1991, 1992; Dupont et al., 1994; Xu, Nolan, & Cole, 1994; Liu et al., 1995; Van Berkel & Zhou, 1995b; McCarley et al., 1998; Van Berkel et al., 1998; Rondeau et al., [Bibr b173],[Bibr b174]; Guaratini et al., [Bibr b58],[Bibr b59],[Bibr b60]; Ochran & Konermann, 2004; Vessecchi et al., 2007; Van Berkel & Kertesz, 2012).

**Figure 9:**
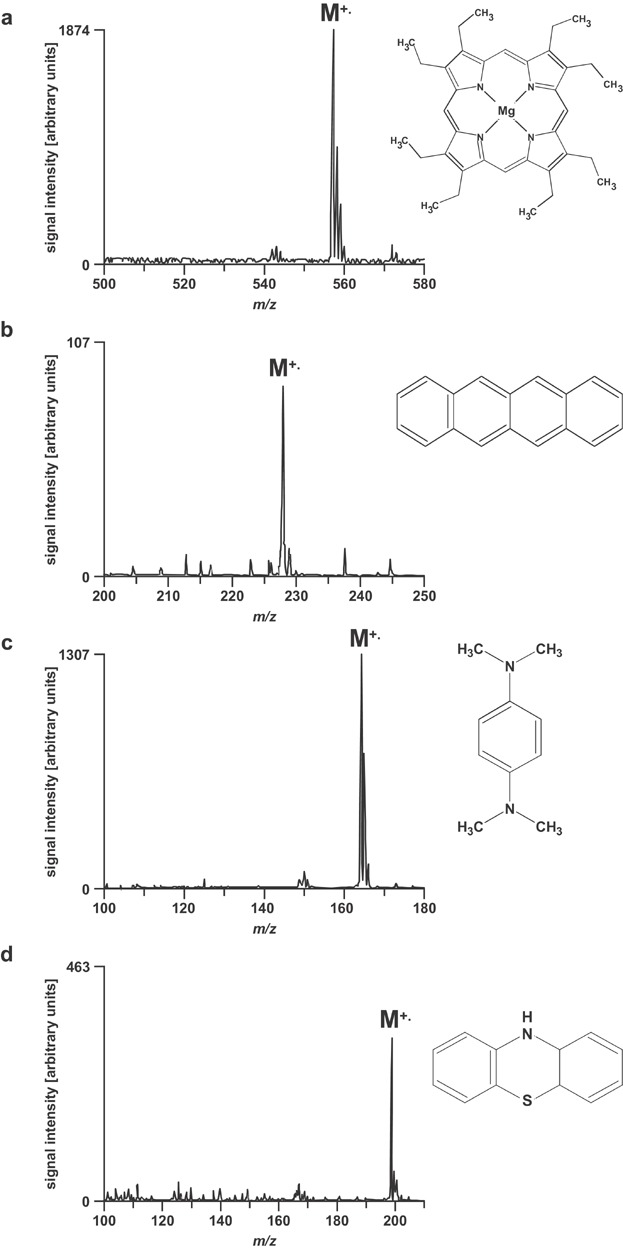
Electrochemical ionization of (a) magnesium octaethylporphyrin, (b) 2,3-benzanthracene, (c) N,N,N',N'-tetramethyl-1,4-phenylendiamine, (d) phenothiazine. Reproduced from Van Berkel, McLuckey, and Glish (1992) with permission of the American Chemical Society (Copyright 1992).

Compounds are converted into radical cations in positive ion mode (Fig. 9) and radical anions in negative ion mode. The types of analytes amenable to electrochemical ionization typically have redox potentials within the following potential limits: −0.8 and +1.0 V versus the saturated calomel electrode (SCE) (Dupont et al., 1994; Van Berkel & Zhou, 1995b). Such compounds are composed of highly conjugated systems and/or contain heteroatoms with lone pair electrons which aid in delocalization of the unpaired electron and positive charge, thereby stabilizing the radical ion (Van Berkel, McLuckey, & Glish, 1992). One requirement for efficient electrochemical ionization is that only the redox reaction involving the analyte of interest should occur at the electrode emitter. Thus, proper choice of solvents, solvent additives and electrode material is very important. Typically, aprotic, nonnucleophilic solvents (e.g., acetonitrile, methylene chloride) in combination with inert electrodes (e.g., Pt) are employed (Schaefer et al., 2007; Vessecchi et al., 2011; Zhang et al., 2012a). Another factor influencing the efficiency of electrochemical ionization is the mass transport to the electrode. Typically, highest signal intensities are observed at low flow rates due to the fact that analytes will have sufficient time to reach the electrode surface by diffusion (Van Berkel & Zhou, 1995b). The mass transport can further be enhanced by optimizing the emitter geometry (Van Berkel, Asano, & Kertesz, 2002; Van Berkel et al., 2004b; Plattner et al., 2012a). For instance, planar flow-by and porous flow-through emitter electrode configurations exhibit efficient mass transport to the electrode surface and provide near 100% oxidation efficiency even at flow rates of several hundred microliters per minute. The third requirement for efficient electrochemical ionization is that the magnitude of the faradaic current should be sufficient for electrochemical conversion of all the analyte passing through the emitter (Van Berkel & Kertesz, 2007). The conductivity of the solvent was identified as important factor influencing the current. Thus, the addition of an ESI friendly electrolyte (e.g., lithium triflate or ammonium acetate) was suggested (Van Berkel & Zhou, 1995b). Alternatively, the faradaic current and, thus, the electrochemical conversion at the emitter electrode can be enhanced by adding an upstream current loop to the ESI source circuit (Konermann, Silva, & Sogbein, 2001; Ochran & Konermann, 2004; Van Berkel et al., 2004b; Van Berkel & Kertesz, 2005). The upstream loop is formed by a ground contact to solution upstream of the emitter electrode. The resulting circuit represents two coupled electrolytic cells that share the ESI capillary as a common electrode (Fig. 0). To some extent, the magnitude of the ground current can be controlled by the electrolyte concentration, by the dimension of the capillary connecting the ground and the emitter electrode, and by the rate of the electrochemical reactions at the ground electrode. The effect of the capillary length on the electrochemical ionization of ferrocene is shown in Figure 11.

**Figure 10:**
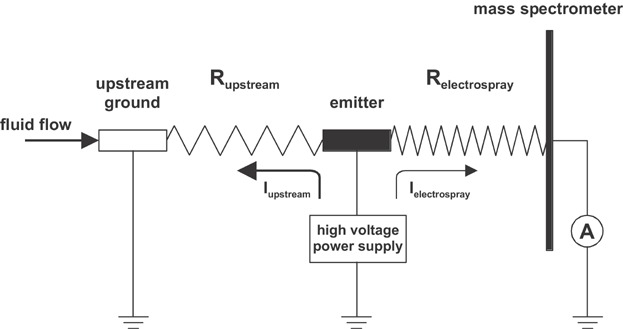
Schematic representation of the electrical equivalent circuit for the ESI source in positive ion mode showing an upstream current loop. Reproduced from Van Berkel et al. (2004b) with permission of the Springer-Verlag (Copyright 2004).

**Figure 11:**
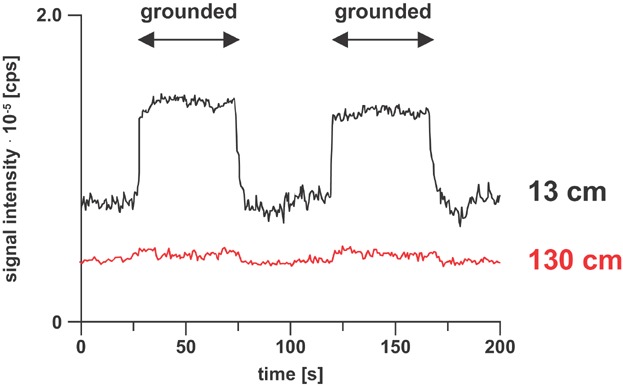
Selected ion chromatograms of ferrocene radical cations (*m*/*z* = 186). The upstream ground was separated from the emitter electrode with a 13 or 130 cm fused silica capillary. Reproduced from Ochran and Konermann (2004) with permission of the Springer-Verlag (Copyright 2004).

## IV COMBINING ELECTROCHEMICAL CELLS WITH ESI-MS

Electrochemical reactions are integral parts of the ESI process. These reactions maintain the quasicontinous production of charged droplets and ultimately gas-phase ions. A conventional ESI source can be seen as a controlled-current cell consisting of two electrodes (Fig. 7). Electrochemical reactions occurring in that cell can be used to analytical advantage. A limitation of this setup is, however, its inability to precisely control the electrochemical potential at the emitter electrode. Thus, particularly for studying mechanistic aspects of electrochemical processes, experimental setups allowing partial or complete separation of the electrochemical processes studied from the ESI-inherent EC were found to be more appropriate. Typically, the analytical cells used are controlled-potential cells.

### A. Solvent Selection for EC/ESI-MS

Proper selection of the solvent system used is of utmost importance for efficient EC/ESI-MS application. In order to obtain good electrochemical conversion, some kind of supporting electrolyte is usually added to increase the conductivity of the sample solution. For aqueous solutions, acids, bases, or salts (e.g., formic acid, acetic acid, ammonia, ammonium formate, and ammonium acetate) are commonly used as supporting electrolyte. To organic solvents, soluble salts (e.g., tetraalkylammonium salts, lithium triflate) are added. High electrolyte concentrations, however, may lead to reduced ionization efficiency in ESI and should therefore be avoided. Likewise, non-volatile salts that are commonly used for EC (i.e., phosphate, sulfate, borate additives) are not suitable for ESI. Obviously, solvent systems for both techniques cannot be independently optimized. Usually, a compromise in terms of proper selection of the kind of supporting electrolyte, its concentration as well as the pH of the solution has to be found to enable the efficient on-line coupling of EC to ESI-MS.

### B. Decoupling of the Electrochemical Cell From the ESI Circuit

The coupling of the two electrochemical circuits part of an EC/ESI-MS system is complicated by the need to decouple the analytical cell from the ESI high voltage (Zettersten et al., 2006). Decoupling is necessary for proper control of the electrochemical potential at the working electrode, and is usually accomplished either by using sufficiently long transfer lines (∼30 cm) between the electrochemical cell and the ESI source (Zhou & Van Berkel, 1995; Van Berkel & Zhou, 1995a), the insertion of a ground point between the two cells (Zhou & Van Berkel, 1995), or by allowing the whole system to float on the potential induced by the ESI high voltage (Zhou & Van Berkel, 1995; Xu, Lu, & Cole, 1996; Lu, Xu, & Cole, 1997; Zhang et al., 2002; Bökman et al., 2004; Zettersten et al., 2006). The disadvantage of using long transfer lines is the increased response time, which makes the detection of unstable intermediates or products of electrochemical reactions difficult. At ground points, secondary electrochemical reactions may occur that would alter the analyte of interest with only limited user control (Konermann, Silva, & Sogbein, 2001; Ochran & Konermann, 2004; Van Berkel & Kertesz, 2005). The main problem of floating is the hazard for the hardware and the operator associated with the use of high voltages. More appropriate decoupling methods try to physically separate the two electrochemical circuits. One competent approach involves the integration of the analytical electrochemical cell into the autosampler system part of a flow-injection analysis—ESI-MS system (Bökman et al., 2004; Pitterl, Chervet, & Oberacher, 2010). Such a setup consists of two independent flow paths, and the sample is transferred by the injection system. In the setup presented by Chen and coworkers the two electrical circuits were physically separated by using the ambient ionization technique desorption electrospray ionization (DESI) (Li, Dewald, & Chen, 2009; Zhang, Dewald, & Chen, 2011a; Zhang et al., 2011b; Lu et al., 2012; Liu et al., 2012). Besides efficient decoupling, EC-DESI was found to have fast response times, the freedom to choose favorable ionization modes and solvent systems, as well as the absence of any kind of alteration of the mass spectrum resulting from secondary redox reactions possibly occurring in ESI (Benassi et al., 2009).

Physical separation of the site of the electrochemical reaction and the ionization leads to an increase of response time. This can be disadvantageous in analyzing short-lived intermediates or products of electrochemical reactions.

### C. Secondary Electrochemical Reactions

EC/ESI-MS systems contain potential sites for electrochemical reactions with limited user control. Such unwanted secondary reactions can take place at ground points, the ESI source as well as auxiliary and reference electrodes, and should be kept to a minimum (Deng & Van Berkel, 1999a; Bökman et al., 2004; Zettersten et al., 2006). Thus, the mass transport to the electrodes is usually limited by using high solution flow rates, by employing electrodes with small surface areas or by avoiding direct contact of electrodes with the analyte in the flow stream (e.g., separate electrode compartments).

Unwanted secondary reactions can be avoided by using alternative ionization methods. Redox reactions are hardly observed in DESI (Benassi et al., 2009), and DESI has been combined with EC (Li, Dewald, & Chen, 2009; Zhang, Dewald, & Chen, 2011a; Zhang et al., 2011b; Lu et al., 2012; Liu et al., 2012). Furthermore, different kinds of contactless spraying methods have been developed, and they show promise to allow efficient ionization without applying the high voltage directly to the sample solution (Stark et al., 2010; Huang, Li, & Cooks, 2011; Qiao et al., 2012).

**Figure 12:**
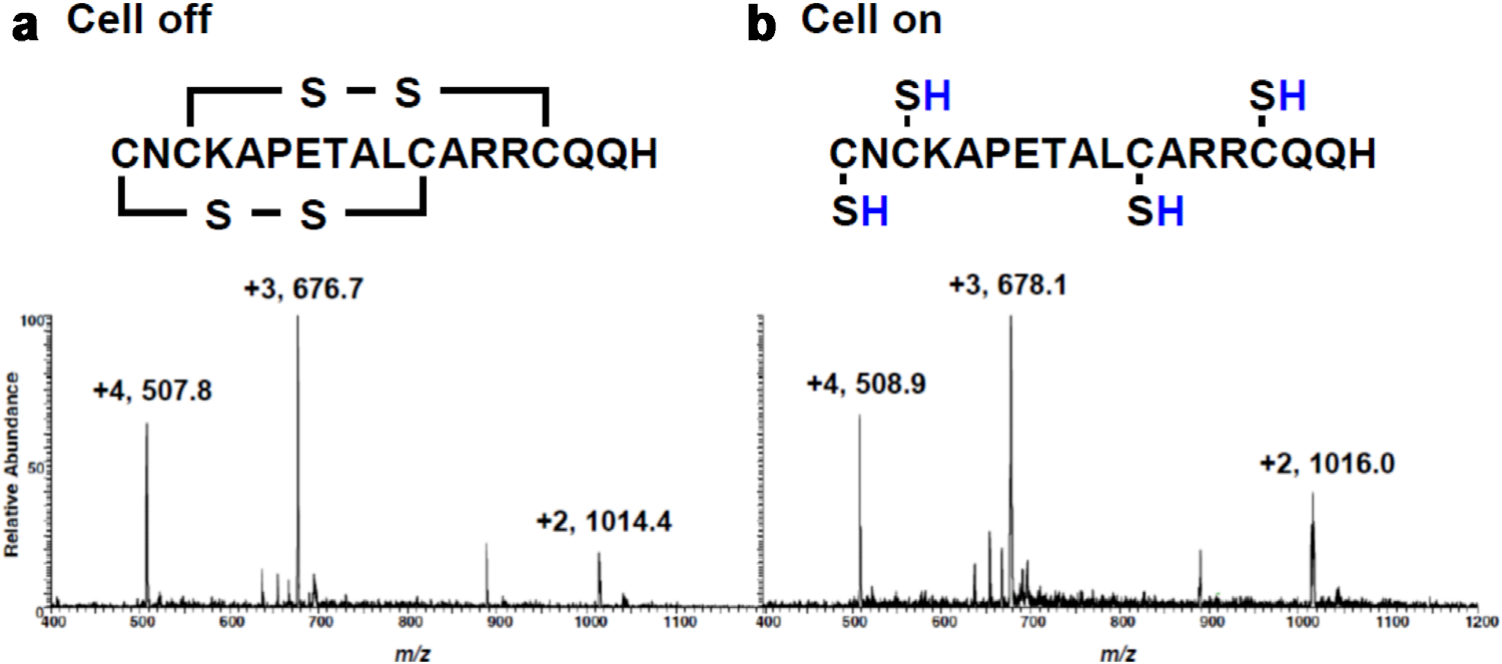
Reductive cleavage of disulfide bonds in apamin. DESI-MS spectra were obtained by analyzing a solution of 20 µM apamin in methanol/water containing 0.5% formic acid. The solution was pumped through an electrochemical cell with an applied potential of (a) 0.0 V and (b) −1.5 V. Reproduced from Lu et al. (2012) with permission of the Springer-Verlag (Copyright 2012).

Chen and coworkers have extensively applied the developed EC/DESI setup to study the reduction of peptides and proteins (Li, Dewald, & Chen, 2009; Zhang, Dewald, & Chen, 2011a; Zhang et al., 2011b; Lu et al., 2012; Liu et al., 2012). Direct reduction of peptides and proteins mainly targets disulfide bond bridges. Disulfide bonds are one of the most common post-translational modifications and provide covalent cross-linkages in native proteins for maintaining the three-dimensional structures of proteins and their biological activities. However, the presence of disulfide linkages increases the complexity for the protein structure elucidation by MS. Accordingly, they need to be reduced, and this can be accomplished by EC. In Figure 12 the electrochemical reduction of apamin is shown. Apamin has 18 amino acid residues in a single peptide chain and carries two knotted disulfide bonds located at Cys^1^–Cys^11^ and Cys^3^–Cys^15^. Figure 12a shows the DESI-MS spectrum acquired when the peptide solution was passed through the electrochemical cell with no potential applied. Applying a negative voltage to the working electrode gave rise to complete cleavage of the disfluide bonds (Fig. 12b).

### D. Instrument Configurations Used in EC/ESI-MS

#### 1. Electrochemical Cells Integrated in ESI Emitters

**Figure 13:**
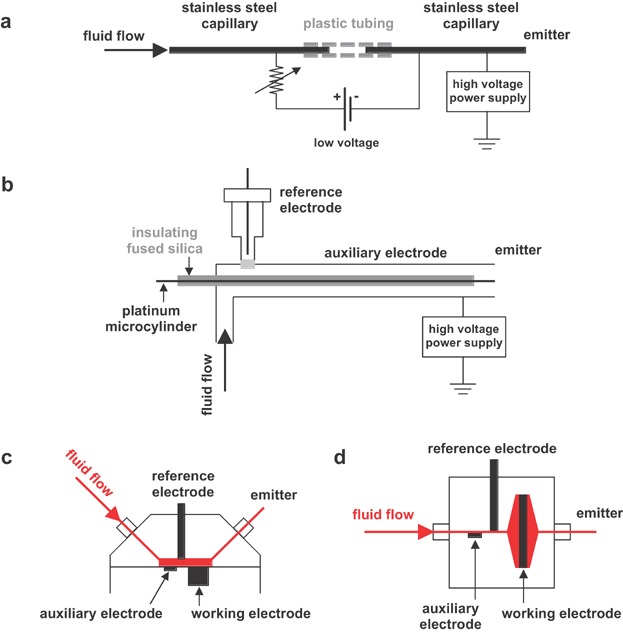
Schematic diagrams of (a) a two-electrode electrochemical ESI emitter (Zhang et al., 2002), (b) a three-electrode electrochemical ESI emitter with the high voltage applied to the auxiliary electrode (Xu, Lu, & Cole, 1996), and three-electrode electrochemical ESI emitters with the high voltage applied to (c) a planar working electrode as well as (d) a porous working electrode (Van Berkel, Asano, & Granger, 2004a).

One way of combining EC with ESI is to integrate the emitter electrode in a controlled-potential electrochemical cell (Fig. 13). A simple setup for incorporating an electrochemical cell into the ESI source was presented by Van Berkel and coworkers (Zhou & Van Berkel, 1995) and Brajter-Toth and coworkers (Zhang et al., 2002; Mautjana et al., 2008a,2008b, 2009; Looi, Eyler, & Brajter-Toth, 2011). The ESI emitter was the working electrode of a two-electrode cell (Fig. 13a). This cell was constructed by connecting the ESI emitter through low volume plastic tubing with the stainless-steel counter/reference electrode. The voltage was applied with a 9 V battery across a variable resistor. Decoupling of the electrochemical cell from the ESI high voltage was accomplished by allowing the EC system to float on the potential induced by the ESI high voltage.

Cole and coworkers have integrated a three-electrode cell into the ESI source (Xu, Lu, & Cole, 1996; Lu, Xu, & Cole, 1997). In the three-electrode cell, the working electrode was a platinum wire covered by insulating fused silica (Fig. 13b). The fused-silica capillary prevented electrical contact between the working electrode and the sample solution until immediately prior to the electrospray region. Furthermore, it insulated the working electrode from the stainless-steel auxiliary electrode, which also functioned as the ESI emitter electrode. The reference electrode was placed upstream outside of the sprayer in a separate compartment. The voltage was applied with a potentiostat and was floated at the ESI voltage. The most important feature of the developed setup was that it generated electrochemical intermediates (e.g., radical cations) and products in situ at the tip of the ESI emitter, thus keeping response times to a minimum.

Van Berkel and coworkers have also incorporated three-electrode cells into the electrospray emitter circuit (Van Berkel, Asano, & Granger, 2004a; Kertesz, Van Berkel, & Granger, 2005). Two different basic cell designs were used, namely, a planar flow-by working electrode (Fig. 13c) and a porous flow-through working electrode design (Fig. 13d), each operated with a potentiostat floated at the ESI voltage. In each case the working electrode also functioned as the emitter electrode of the ESI source. Reserpine was used as sample to test the cells. The authors showed that reserpine oxidation was tunable by the electrochemical potential applied. Extensive reserpine oxidation was observed at potentials more positive than the potential necessary to induce reserpine oxidation. This oxidation occurred at the working electrode. Unexpectedly, reserpine oxidation was also observed at very negative potentials. This unwanted analyte electrolysis occurred at the auxiliary electrode and was prevented either with the auxiliary electrode removed from direct contact with the analyte in the flow stream, or with mass transport of the analyte to that electrode limited by another method (e.g., small surface area).

#### 2. Hyphenation of Discrete Systems

**Figure 14:**
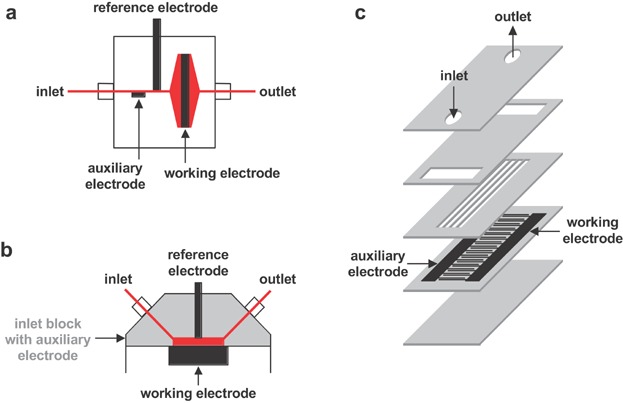
Schematic diagrams of electrochemical cell designs used for EC/ESI-MS: (a) a three-electrode cell with a porous flow-through working electrode, (b) a three-electrode cell with a planar flow-by working electrode, and (c) a microfluidic two-electrode cell in a lab-on-chip format. Reproduced from Mengeaud et al. (2002) with permission of the Royal Society of Chemistry (Copyright 2002).

Another way of combining EC with ESI is to hyphenate discrete systems (Fig. 14). In such a setup ESI-MS is employed to specifically detect and characterize the products of electrochemical reactions produced in an independently working electrochemical cell.

The simplest electrochemical cell hyphenated to ESI-MS consisted of two electrodes only (Bond et al., 1995). In such a flow cell, two tubular electrodes were separated by insulating tubing (e.g., PEEK, Teflon). The setup was successfully applied to induce oxidation of copper, nickel and cobalt diethyldithiocarbamates. A similar two-electrode setup was used to study the electrochemical reduction of metalloproteins (Johnson et al., 2001).

In state-of-the-art EC/ESI-MS, three-electrode cells are hyphenated to ESI-MS. In one commonly applied cell design a porous flow-through working electrode is used (Fig. 14a). This cell design has been introduced to EC/MS by Brajter-Toth and coworkers (Volk et al., 1988; Volk, Yost, & Brajter-Toth, 1989, 1992). In this pioneering work, thermospray was used as ionization technique, and the redox reactivity of uric acid, 6-thioxanthine and two purines was studied at a glassy carbon working electrode. The porous-electrode flow cell has been commercialized by ESA, Inc. (Bedford, MA) which is now part of Thermo Fisher Scientific (Waltham, MA).

Cells with porous electrodes are considered to provide good conversion rates even at high flow rates due to the large surface area provided. A further characteristic of these coulometric cells is the low maintenance effort. The electrodes are usually simply cleaned by flushing with appropriate solvents. Even though adsorption can take place on the electrode surface and residues might not be fully removed, the effects on the oxidation process often remain negligible (Baumann & Karst, 2010). Sometimes, however, life history and/or age of the electrochemical cell could have an impact on the oxidation reactions observed (Permentier & Bruins, 2004).

**Figure 15:**
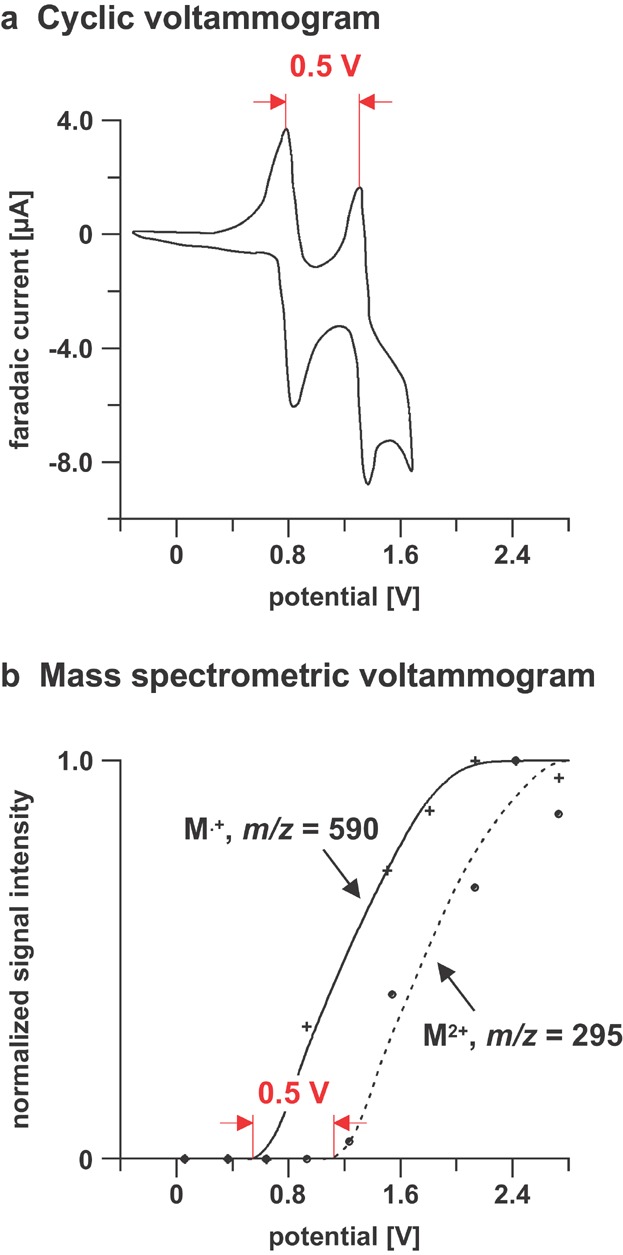
a: Cyclic voltammogram and (b) mass spectrometric voltammogram of nickel(II) octaethylporphyrin. Reproduced from Zhou and Van Berkel (1995) with permission of the American Chemical Society (Copyright 1995).

The first EC/ESI-MS setup using a porous electrode was presented by Van Berkel and coworkers (Zhou & Van Berkel, 1995), and was applied to study the oxidation of nickel(II) octaethylporphyrin. The cyclic voltammogram of this compound and the mass spectrometric voltammograms of the oxidation products are shown in Figure 15. These diagrams clearly indicated that nickel(II) octaethylporphyrin undergoes two reversible one-electron oxidation reactions giving rise to the corresponding monocation (M^•+^, *m*/*z* = 590) and dication (M^2+^, *m*/*z* = 295). The observed differences in the “appearance potentials” were 0.5 V.

Bruins and coworkers have extensively used three-electrode cells with the porous electrode to study the redox reactivity of drugs (Jurva, Wikstrom, & Bruins, 2000; Jurva et al., 2003). This work laid the foundation for the use of EC/ESI-MS techniques to mimic phase I oxidative reactions in drug metabolism. EC was found to be particularly useful in cases where the P450 enzyme catalyzed reactions are supposed to proceed via a mechanism initiated by a one-electron oxidation, such as N-dealkylation, S-oxidation, P-oxidation, alcohol oxidation, and dehydrogenation. As valuable information concerning the sensitivity of a substrate towards oxidation can be obtained from EC/ESI-MS experiments, the technique is regarded as an efficient tool in the drug development process.

**Figure 16:**
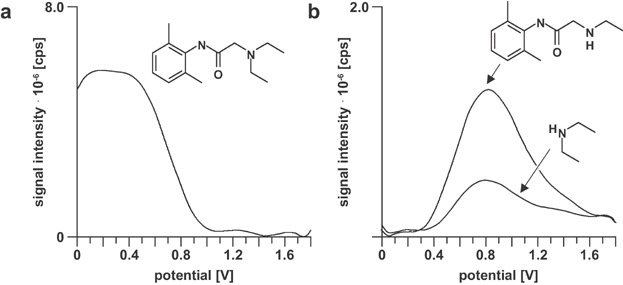
Extracted ion voltammograms of [M + H]^+^ ions of (a) lidocaine and (b) its oxidation products plotted against the applied cell potential. Reproduced from Jurva, Wikstrom, and Bruins (2000) with permission of John Wiley & Sons (Copyright 2000).

One of the first examples of successful mimicking of drug metabolism is shown in Figure 16. In a proof-of-principle study, Bruins and coworkers have applied EC to oxidize lidocaine (Jurva, Wikstrom, & Bruins, 2000). Two different oxidation products were detected by ESI-MS. Of particular importance was the formation of dealkylated lidocaine, because this compound is also formed *in vivo* catalyzed by CYP3A4.

**Figure 17:**
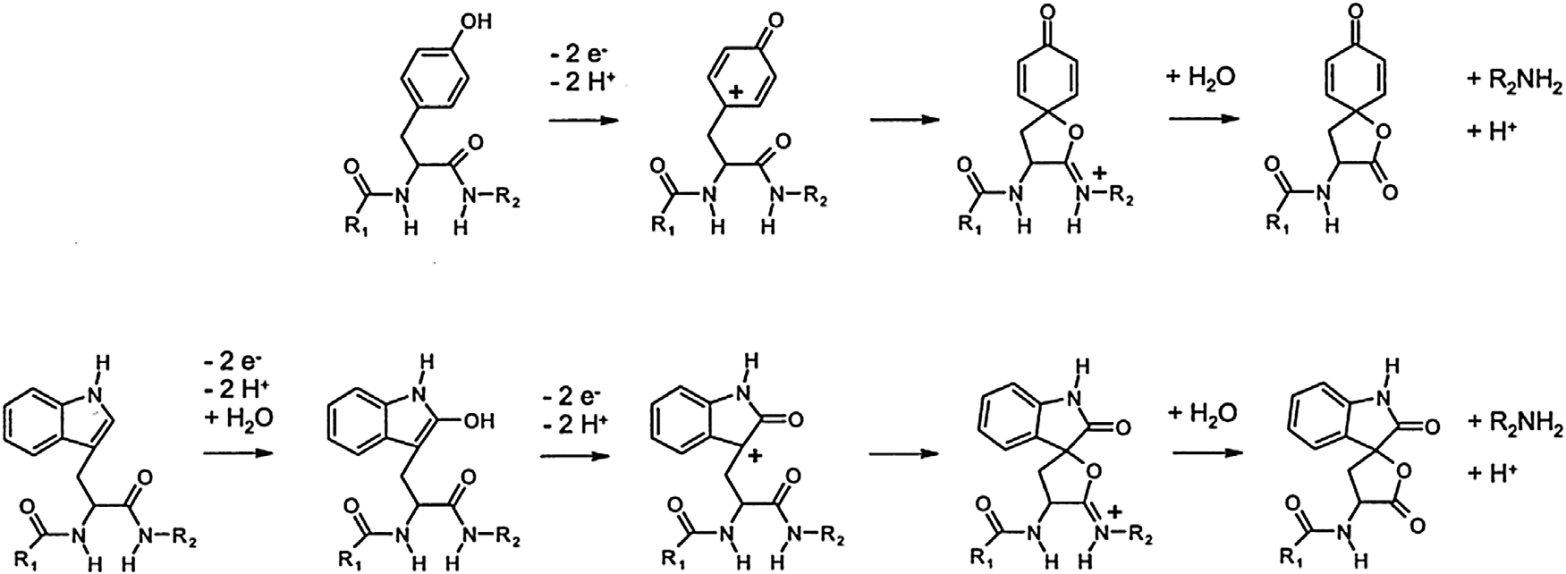
Proposed reaction mechanism for electrochemical oxidation and cleavage at tyrosine (top) and tryptophan (bottom). *R*_1_ and *R*_2_ are the parts of the protein N-terminal and C-terminal, respectively, to tyrosine and tryptophan. Reproduced from Permentier and Bruins (2004) with permission of the Springer-Verlag (Copyright 2004).

Another important application of EC/ESI-MS introduced by Bruins and coworkers using the porous electrode system was oxidative cleavage of peptides and proteins (Permentier et al., 2003; Permentier & Bruins, 2004; Roeser et al., 2010b). Generally, oxidation of peptides and proteins involves the amino acids tyrosine, tryptophan, cysteine, methionine, and histidine. The electrochemical oxidation of tyrosine- or tryptophan-containing species gives further rise to backbone cleavage at the C-terminal side of these specific amino acids (Fig. 17). Due to its distinct amino acid specificity, its speed of analysis, its easy coupling to MS, EC was considered a potential instrumental alternative to chemical and enzymatic cleavage with immediate applicability in proteomics.

Another important cell type commonly used in EC/ESI-MS is based on the planar flow-by working electrode design (Fig. 14b). This cell design has been introduced to EC/ESI-MS by Van Berkel and coworkers to study the oxidation reaction of β-carotene on a glassy carbon electrode (Zhou & Van Berkel, 1995). Thin-layer cells are commercially available from Bioanalytical Systems, Inc. (West Lafayette, IN), Antec (Zoeterwoude, The Netherlands), or Kimoto Electric Co (Osaka, Japan). In comparison to porous electrodes, the surface area of planar electrodes is rather low, and the flow rate can be used to efficiently control the conversion rate (Erb et al., 2012). For obtaining very high conversion rates the cells are usually operated at very low flow rates (<10 µL/min). Another parameter that might influence the conversion efficiency of this kind of electrochemical cell is the analyte concentration. Within the residence time the analyte needs to migrate to the electrode to become oxidized. Theoretically, the more analyte available, the more analyte should be converted. It seems, however, that a maximum conversion efficiency exists for thin-layer cells (Erb et al., 2012). The electrochemical cell can be overloaded. As half-wave potentials and peak potentials, which are often used as specific parameters to characterize redox systems, can be shifted to higher values with increasing analyte concentrations, loading effects can have particular implications for mechanistic studies. A clear advantage of thin-layer cells is the possibility to use different electrode materials. Besides glassy carbon, platinum and boron-doped diamond electrodes are the most promising alternatives. Usually, less adsorption on these electrode materials occurs (Baumann & Karst, 2010). If adsorption residues are observed, they can be manually polished from the surface. In some cases analyte adsorption can be of analytical advantage. Van Berkel and coworkers were the first that demonstrated the usefulness of electrochemically controlled sample preconcentration and purification prior to ESI-MS analysis (Pretty et al., 2000). Tamoxifen and its metabolite 4-hydroxytamoxifen were accumulated on a glassy carbon electrode via nonelectrolytic adsorption. Once on the electrode, the analytes were washed free of sample matrix. For release and subsequent mass spectrometric detection of the unaltered analytes, the potential of the working electrode was changed. With the developed method, nanomolar levels of the analytes were detected in urine samples. Nyholm and coworkers took advantage of nonelectrolytic adsorption for the detection of thiols (Bökman et al., 2004). Thiols are known to strongly adsorb on gold surfaces, and this was used to preconcentrate 1-hexenthiol on a gold working electrode. Desorption was made by applying an electrochemical potential that was sufficiently high to induce oxidation of the thiols to the corresponding sulfinates and sulfonates. These species were detected by ESI-MS. More recently, Lev and coworkers presented another approach for electrochemical preconcentration of analytes (Gutkin, Gun, & Lev, 2009). Their approach was based on the electrodeposition of an active silver layer, subsequent specific accumulation of the target analyte onto the active layer, and finally oxidative electrostripping of the conductive layer along with the supported analyte to ESI-MS. The technique was found to be useful for the analysis of homocysteine and other organothiols.

When using a thin-layer cell, electrode cross-talk may become a problem (Deng & Van Berkel, 1999a; Bökman et al., 2004; Zettersten et al., 2006). The counter and working electrodes are often positioned so close to each other that only a thin spacer, defining the flow channel, separates them. In such an arrangement, where the solution containing the analytes passes over both the working and auxiliary electrodes, there is a risk that electrochemical reactions at the auxiliary electrodes may effect the appearance of the mass spectra. One such possibility involves redox cycling, by which the product formed at the working electrode undergoes a reverse reaction on the counter electrode. Another possibility is that species directly or indirectly formed by electrochemical reactions at the auxiliary electrode may appear in the mass spectra. Such interference, however, can be avoided by using a cell with discrete compartments for working, auxiliary, and reference electrodes.

Another cell type used in EC/ESI-MS is based on microfluidic devices. These chip cells have been introduced by Girault and coworkers (Mengeaud et al., 2002), and they are commercially available from Antec and ALS (Tokyo, Japan). In one setup used the microfluidic electrochemical device consisted of arrays of interdigitated electrodes (Mengeaud et al., 2002; Liu et al., 2012). A schematic representation of this chip is provided in Figure 14c. Nyholm and coworkers integrated gold microcoil electrodes into their chip system (Liljegren et al., 2005). Odijk and coworkers designed an on-chip three-electrode electrochemical cell (Odijk et al., [Bibr b146],[Bibr b147],[Bibr b148]). The microfluidic cells were successfully applied to study the redox reactivity of different drug compounds.

Microfluidic cells in a lab-on-chip format can provide specific advantages. By miniaturization, the surface-to-volume ratio is increased giving rise to improved mass transport properties necessary to achieve a high conversion efficiency of introduced analytes. Furthermore, cell volumes are small, giving the possibility to work with small volumes and amounts of sample. The miniaturized cells can easily be combined with ESI emitters. Such integrated systems offer fast response times (<1 sec), even though they are operated at low flow rates (Liljegren et al., 2005). Also, the lab-on-chip format may allow the production of low-cost disposables to circumvent the need for extensive cleaning of the electrodes after use.

### E. EC/ESI-MS Applications

Important fields of applications of EC/ESI-MS are summarized in Table2. The technology seems to be particularly useful for simulating drug metabolism reactions, for surveying redox processes involving small (bio)organic molecules, and for studying the cleavage of amide and disulfide bonds in peptides.

**Table 2:** Important applications of coupled with LC/ESI-MS

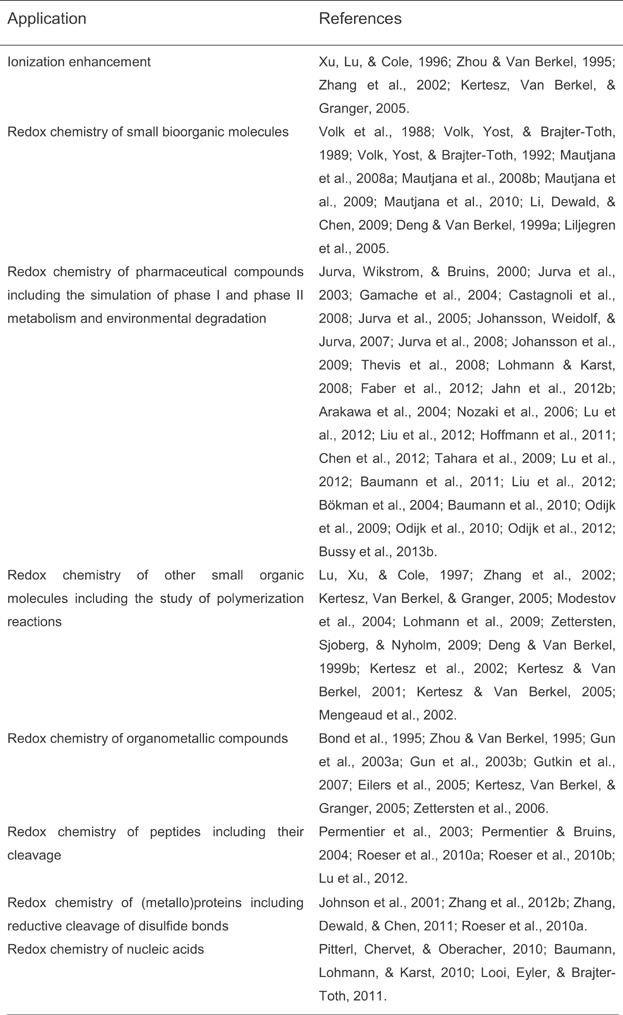

## V. COMBINING ELECTROCHEMICAL CELLS WITH LC/ESI-MS

ESI-MS represents a powerful method to characterize the products of electrochemical reactions. The analytical power of EC/ESI-MS can further be increased by integrating LC as an additional dimension of separation. Chromatographic separation is particularly useful to reduce the complexity of the sample submitted either to the EC cell or to ESI-MS. Due to its compatibility with EC and ESI, reversed-phase chromatography is the preferred chromatographic mode of operation. Usually, separations are accomplished on C8 or C18 columns. The inner diameters (i.d.) of the columns are typically ranging from 2 to 4.6 mm. In some cases miniaturized columns with i.d. of 100–200 µm have been applied (McClintock, Kertesz, & Hettich, 2008; Erb et al., 2012; Plattner et al., 2012b). Mixtures of organic solvents (i.e., acetonitrile and methanol) with volatile acids or salts (i.e., formic acid, ammonium format, ammonium acetate) are used as mobile phases. EC experiments have been performed either in three-electrode cells with porous flow-through working electrodes, in three-electrode cells with planar flow-by working electrodes or in microfluidic three-electrode cells in a lab-on-chip format.

### A. Instrument Configurations Used in EC/LC/ESI-MS

**Figure 18:**
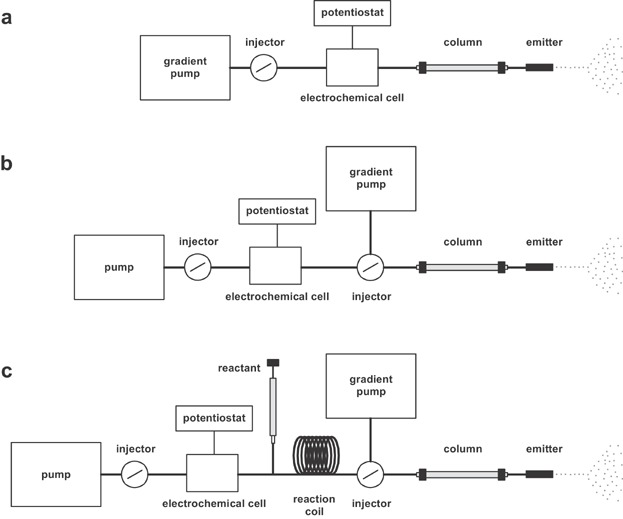
Schematic diagrams of different EC/LC/ESI-MS configurations. (a) The electrochemical cell is located between the injector and the column. (b) The electrochemical cell is part of a flow-injection system which delivers sample to the injection port of the LC/ESI-MS system. (c) The flow-injection system is extended by a reagent delivery system and a reaction coil to enable trapping of oxidation products.

Different instrumental configurations were presented enabling EC/LC/ESI-MS (Fig. 18). LC/ESI-MS is used to characterize products of electrochemical reactions. The EC cell can either be run off-line or can be integrated in the LC/ESI-MS system.

#### 1. Off-Line EC/LC/ESI-MS

In off-line experiments, products of electrochemical reactions are collected and subsequently submitted to LC/MS analysis. A clear advantage of off-line EC/LC/ESI-MS is the separation of EC and ESI-MS. Thus, experimental conditions for both methods can be optimized almost independently allowing the use of high concentrations of non-volatile buffer additives for EC (e.g., phosphate, perchlorate, chloride) that would be incompatible with on-line mass spectrometric detection (Li et al., 1999; de Lima, Bonato, & da Silva, 2003; Madsen et al., 2007, 2008aa,2008b; Nouri-Nigjeh et al., 2010, 2011b,2011c). Another advantage of the off-line system is the possibility of using extended reaction times giving rise to increased electrochemical conversion. Moreover, in the off-line approach any type of electrochemical cell can be sampled. Thus, diverse electrode materials can be used for electrochemical experiments. To reduce adsorption on electrodes, often high amounts of organic solvents (i.e., acetonitrile, methanol) are added to the sample solution (Nouri-Nigjeh et al., 2011b, 2011c; Jurva et al., 2008).Nouri-Nigjeh et al., 2011b,c

#### 2. On-Line EC/LC/ESI-MS

The beauty of the on-line approach is the integration of EC in the LC/ESI-MS setup. Thus, one integrated instrument is capable to perform redox reactions and to subsequently separate, detect and characterize the reaction products formed. In the simplest setup used (Fig. 18a), the electrochemical cell is integrated in the LC system between the injector and the chromatographic column (Iwahashi & Ishii, 1997; Iwahashi, 1999; Gamache et al., 2004; Lohmann & Karst, 2006; Tahara et al., 2007; Karady et al., 2011). Thus, samples enter the electrochemical cell after injection. As the commonly applied chromatographic systems are operated at flow rates of several hundred microliters per minute, cells with porous flow-through working electrodes are exclusively used for such type of experiment. A broader range of cell designs and, thus, electrode materials becomes applicable by combining an independently working EC system with LC/ESI-MS (Fig. 18b). Such an EC system typically consists of an electrochemical cell and a flow-injection analysis system (i.e., a combined pump and injection system) for delivering the sample. This EC system can be operated at very low flow rates giving rise to increased conversion efficiency even by using cells with planar flow-by working electrodes. The samples are injected into the LC/ESI-MS system after passing through the electrochemical cell.

**Figure 19:**
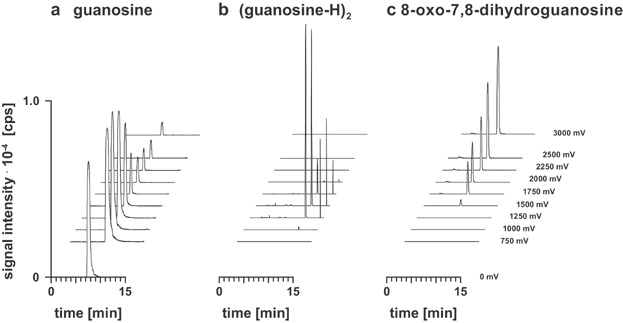
Extracted ion chromatograms of (a) guanosine and its major oxidation products (b) (guanosine-H)_2_ and (c) 8-oxo-7,8-dihydroguanosine obtained at different oxidation potentials. E, 0–3,000 mV; flow rate through the EC cell, 2.5 µL/min; working electrode material, boron-doped diamond; column, Eurospher C18, 5 µm, 200 × 0.2 mm id; mobile phase, (A) 10 mM ammonium formate, pH 7.3, (B) 10 mM ammonium formate containing 50% acetonitrile (v/v), pH 7.3; linear gradient, 5–60% (B) in 10 min; flow rate, 2.5 µL/min; scan, 50–700; sample, 100 µM guanosine dissolved in 10 mM ammonium formate, pH 7.3. Reproduced from Erb et al. (2012) with permission of the Wiley-VCH Verlag (Copyright 2012).

A clear advantage of using LC/ESI-MS to monitor electrochemical processes is the ability to dinguish isobaric species via chromatographic separation. Recently, Oberacher and coworkers demonstrated in EC/ESI-MS experiments that (guanosine-H)_2_ is produced during guanosine oxidation (Pitterl, Chervet, & Oberacher, 2010). More recently, the same group could show that two different isomeric forms of the dimer were generated (Fig. 19), and this was only possible due to chromatographic separation prior to ESI-MS detection (Erb et al., 2012).

An interesting modification of the EC system recently presented by Karst and coworkers involved the addition of a second electrochemical cell (Lohmann et al., 2009). The first cell is run in oxidative mode, and the second cell in reductive mode. Consecutive oxidation and reduction steps were found to induce transformation reactions that are not accessible by oxidation alone (Nouri-Nigjeh et al., 2011b).

Supporting electrolytes typically applied for EC are low concentrations of acids, bases or salts (e.g., formic acid, acetic acid, ammonia, ammonium formate, and ammonium acetate). To avoid adsorption on working electrodes, organic solvents (i.e., acetonitrile, methanol) are usually added to the sample solution.

One clear disadvantage of any EC/LC/ESI-MS is the inability to directly detect short-lived intermediates, as they are not stable products during the chromatographic run. Indirect detection is enabled by trapping. Commonly applied trapping agents for electrophilic compounds include thiols (i.e. cysteine or glutathione to mimic phase II metabolism) and proteins (Getek et al., 1989; Lohmann & Karst, 2006; Lohmann, Hayen, & Karst, 2008). The trapping agent can be added to the sample before or after electrochemical activation. For the automated generation of conjugates, Karst and coworkers have integrated a reagent delivery system and a reaction coil into the EC/LC/ESI-MS system (Fig. 18c). Chromatographic separations allowed the identification of isomeric species indicating that different sites of the tested molecules were oxidatively activated.

A competent approach to increase the amount and specificity of the analytical information accessible by EC/LC/ESI-MS experiments involves the integration of additional detection methods. Circular dichroism detection was found to be beneficial in studying the redox reactivity of chiral drugs. Tahara and coworkers used this technique to distinguish the two diastereomers 9α- and 9β-methoxy-α-tocopherol produced by electrochemical oxidation of (±)-α-tocopherol (Tahara et al., 2008). Radioactivity detection was applied to the selective detection of compounds bearing a radioactive atom. Karst and coworkers have applied EC/LC/ESI-MS with parallel radioactivity detection to identify the main metabolites of a radioligand in mouse body fluids (Baumann et al., 2011). For testing the biological activity of the products of electrochemical reactions, bioaffinity assays were used in parallel to ESI-MS detection. Kool and coworkers integrated an assay indicating binding to the p38α kinase into their EC/LC/ESI-MS system (Falck et al., 2012). Competition of the electrochemical conversion products with a tracer (SKF-86002) that showed fluorescence enhancement in the orthosteric binding site of the p38α kinase was the readout for bioaffinity. A clear advantage of the integration of direct affinity assessment is the possibility to identify active molecules among the plurality of electrochemical conversion products. The electrochemical conversion of 1-{6-chloro-5-[(2R,5S)-4-(4-fluorobenzyl)-2,5-dimethylpiperazine-1-carbonyl]-3aH-indol-3-yl}-2-morpholinoethane-1,2-dione, for instance, resulted in eight products, three of which showed bioaffinity in the p38α bioaffinity assay. Such information might be helpful in drug development to determine critical positions of a molecule influencing its affinity towards the targeted protein and, thus, to find more potent ligands.

### B. Instrument Configurations Used in LC/EC/ESI-MS

The electrochemical cell can be integrated post-column into an LC/ESI-MS system. As the commonly applied chromatographic systems are operated at flow rates of several hundred microliters per minute, electrochemical cells with porous flow-through working electrodes are exclusively used for such type of experiment. A known problem in LC/EC/ESI-MS is analyte adsorption on the glassy carbon electrodes which can have a negative impact on chromatographic performance indicated by peak broadening and tailing (van Leeuwen, Hayen, & Karst, 2004). Adsorption can be reduced by using mobile phases with high contents of organic solvents.

A clear advantage of LC/EC/ESI-MS is its ability to electrochemically convert species after chromatographic separation. Accordingly, the redox reactivity of selected compounds within complex mixtures can be studied.

Tong et al. (2010) applied LC/EC/ESI-MS in a drug metabolism study. EC was used to mimic oxidative metabolism occurring in vivo. Furthermore, a new approach for the quantification of instable drug metabolites in the absence of any reference standard was presented. The authors demonstrated that indirect quantification of an instable compound will be enabled by EC if the precursor is quantitatively converted into the targeted compound.

Sjöberg and coworkers used LC/EC/ESI-MS to identify antioxidants. By separation of the sample components in a mixture using LC prior to their oxidation in an EC cell and correlation of the retention times obtained with the EC and MS detectors, information, such as antioxidant activity (oxidation potential), capacity (amount), and structural information, regarding individual antioxidants was obtained. The developed LC/EC/ESI-MS method was used to determine the antioxidant activity of polyphenolic compounds (e.g., cathechin, kampferol, resveratrol, and quercetin) in complex samples such as yellow onion extracts by varying the EC cell potential while monitoring the intensities for the compounds studied.

The on-line electrochemical conversion of analytes represents a convenient approach to improve or even enable their detectability in ESI-MS. Karst and coworkers took advantage of this LC/EC/ESI-MS feature to develop methods for the analysis of phenothiazines (Hayen & Karst, 2003) and polycyclic aromatic hydrocarbons (van Leeuwen, Hayen, & Karst, 2004). Chen and coworkers used LC/EC/ESI-MS to analyze anilines (Chen et al., 2006), and Arakawa and coworkers to analyze zotepine (Nozaki et al., 2009). In all cases detection sensitivity was significantly enhanced by electrochemical oxidation of the targeted analytes.

Another approach developed to improve or enable detection of compounds with ESI-MS involves conjugation with electrochemically active species. The formed derivatives are efficiently detected after electrochemical oxidation. Conjugation with ferrocene-containing compounds was introduced by Van Berkel et al. (1998) as a method for the analysis of alcohols, sterols and phenols. The ferrocene derivatives were converted into the molecular radical cations by oxidation within the ESI source. The utility of this derivatization approach for the selective detection of alcohols was demonstrated using a saw palmetto fruit extract that contained a variety of alcohols at low levels. The combination of conjugation with subsequent LC/EC/ESI-MS analysis was first introduced by Diehl, Liesener, and Karst (2001). This approach was found to be particularly useful for the analysis of complex mixtures. The ferrocene derivatives were separated by LC, and detected by ESI-MS after oxidation in the electrochemical cell. Ferrocene-based reagents were developed for specific conjugation to alcohols, phenols, isocyanates, and cysteine-containing proteins (Diehl, Liesener, & Karst, 2001; Diehl & Karst, 2002b; Diehl et al., 2004; Seiwert, Henneken, & Karst, 2007; Seiwert & Karst, 2008).

### C. Applications for Methods Integrating EC Into LC/ESI-MS

Important applications are summarized in Table3. The hyphenation of EC and LC/ESI-MS seems to be particularly useful for studying redox processes involving small bioorganic molecules such as pharmaceutical compounds, environmental pollutants and metabolites.

**Table 3:** Important applications of EC coupled with LC/ESI-MS

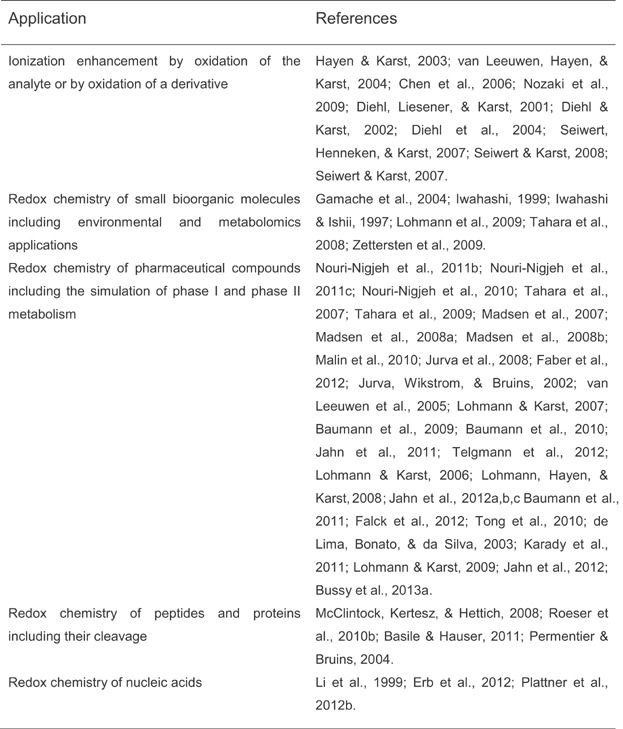

## VI. SUMMARY AND OUTLOOK

MS is a versatile method to monitor and study processes in electrochemical cells. DEMS is used for the analysis of highly volatile compounds such as CO, CO_2_, H_2_, and O_2_. These compounds are produced in batteries, fuel cells, and photovoltaic cells, and they are able to pass through the microporous PTFE membrane representing the interface between CE and MS. In DEMS, ionization is accomplished by EI. DEMS is perfectly suited to analyze processes involving a few compounds only. Due to the lack of any separation step prior to the mass spectrometric detection, the applicability to more complex reactions is limited. Important innovations have been the development of thin-layer cells and the construction of miniaturized interfaces. They allow monitoring processes on smooth electrodes. Currently, the use of DEMS is limited to a few specialized labs that are able to setup tailored instruments for the intended research application. To enable a more widespread use of the technology, the availability of commercial DEMS instrumentation would be obligatory.

ESI-MS is the method of choice for monitoring non-volatile compounds involved in electrochemical processes. Similarly to DEMS, ESI-MS should only be used if a limited number of compounds will be monitored. For the analysis of more complex samples, LC should be integrated. The chromatographic separation can be accomplished either before or after EC.

ESI is an electrochemical process itself, and the EC of ESI can be used for analytical advantage. Redox reactions enabled the ionization of compounds composed of highly conjugated systems and/or contain heteroatoms with lone pair electrons which aid in delocalization of the unpaired electron and positive charge, thereby stabilizing the produced radical ion. As the electrochemical ionization only works well at very specific experimental conditions, this technique has gained only little importance.

For monitoring processes in electrochemical cells with ESI, the inherent EC of ESI should be suppressed to avoid alteration of the experimental results observed. For proper control of the electrochemical potential at the working electrode of the electrochemical cell, the cell circuit should be decoupled from the ESI circuit. Furthermore, secondary reactions occurring at ground points and at the ESI emitter should be kept to a minimum. The technical implementation of these specifications is not trivial. In comparison to DEMS, however, commercial instrumentation is available. With this support, the first steps into EC/ESI-MS are usually quite easily taken.

Based on the number of publications, the most important field of application of EC/ESI-MS is studying the redox chemistry of pharmaceutical compounds. EC/ESI-MS techniques can be used to mimic phase I oxidative reactions in drug metabolism. Valuable information concerning the sensitivity of a substrate towards oxidation can be obtained from such experiments. Clear advantages of the technique over other *in vitro* methods are the possibility of automation and the speed of analysis. Despite considerable success, due to the fact that EC might overestimate or underestimate the range of *in vivo* drug metabolites, the technique is mainly used for pre-screening purposes complementing other *in vitro* methods.

Other small molecular applications, where EC/ESI-MS holds a lot of promise, are metabolomics and environmental research. To find acceptance, however, the technology needs to be converted into a turnkey solution. Integrated systems for combining EC and ESI-MS are already available. There is, however, still a strong need for defining a set of experimental conditions covering different kinds of electrode materials and solvent additives that enable the efficient transformation of a large variety of small molecules containing different kinds of functional groups.

EC/ESI-MS can also be applied to the characterization of peptides and proteins. EC is able to initiate the cleavage of amide and disulfide bonds. Peptide/protein cleavage is based on the electrochemical oxidation of tyrosine and/or tryptophan, and is considered a potential instrumental alternative to chemical and enzymatic cleavage. Disulfide bonds are reductively cleaved. The possibility of on-line disulfide bond reduction can be beneficial for the determination of disulfide bond arrangements or top-down proteomics strategies. Despite considerable success and interest within the proteomics community, the yield and reproducibility of both cleavage reactions need to be increased to find broad acceptance.

DEMS and ESI-MS represent valuable tools for the characterization of electrochemical reactions and have found widespread application. Nevertheless, both methods can only be used to characterize a defined part of the total chemical space. Accordingly, there is a clear trend towards the integration of additional analytical methods into the existing technology. Particularly, different kinds of spectroscopic methods are combined with MS. Very recently, Behm and coworkers have developed an experimental setup for integrating DEMS and ESI-MS monitoring of an electrochemical cell (Zhao, Jusys, & Behm, 2010, 2012). Such setups hold the promise to comprehensively characterize electrochemical processes and to increase our knowledge about the mechanisms involved in the formation of intermediates, products and by-products of electrochemical reactions.

## VII. ABBREVIATIONS

ATR-FTIRS: attenuated total reflection-infrared spectroscopy
DEMS: differential electrochemical mass spectrometry
DESI: desorption electrospray ionization
EC: electrochemistry
EI: electron ionization
EQCM: electrochemical quartz crystal microbalance
ESI: electrospray ionization
i.d.: inner diameter
LC: liquid chromatography
MS: mass spectrometry
RHE: reversible hydrogen electrode
SCE: saturated calomel electrode
WE: working electrode
